# Utilisation of medical rehabilitation services by persons of working age with a migrant background, in comparison to non-migrants: a scoping review

**DOI:** 10.1186/s40985-020-00134-5

**Published:** 2020-08-03

**Authors:** Maria Dyck, Jürgen Breckenkamp, Julia Wicherski, Chloé Charlotte Schröder, Jean-Baptist du Prel, Oliver Razum

**Affiliations:** 1 School of Public Health, Bielefeld University, Bielefeld, Germany; 2 Department of Occupational Health Science, University of Wuppertal, Wuppertal, Germany

## Abstract

In Germany, an ageing population is affected by societal and political changes due to demographic transition, e.g. by a prolonged working life for older employees. Demographic change also influences persons of higher working age with a migrant background. In 2018, 25% of all employees in Germany had a migrant background. Those affected by poor health at a higher working age can benefit from medical rehabilitation services, which aim to prevent early retirement and disabilities. So far, the utilisation of medical rehabilitation has been lower among persons of foreign nationality (often the only available proxy for migrant background), compared to that of Germans. The aim of this scoping review is to assess the utilisation of medical rehabilitation services by those with migrant background (PMB) and those without (non-PMB) and to identify the differences between these groups. We included 25 studies in our analysis, which were mainly secondary analyses of routine data and also a small number of primary studies. The results were inconsistent: studies published before 2018 showed a lower use of rehabilitation services for persons of foreign nationality compared to Germans. However, no differences were found between PMB and non-PMB in studies published in 2018 or later. PMB, as well as foreign nationals, showed poorer health before medical rehabilitation utilisation and had a higher chance of occupational disease and a lower education level. We identified a lower work-related performance, as well as barriers (e.g. information deficits) in the utilisation of rehabilitation services for groups of PMB. Our review is limited in that we cannot generalise our results to all PMB living in Germany. This is because of the heterogeneity, the limited number of studies and lack of representativeness in some studies. In many cases, studies only analyse the nationality, but they lack information about the second generation PMB. Future studies should survey the utilisation of medical rehabilitation services by migrant background rather than by nationality and focus on changes in the provision of rehabilitation measures following diversity-centred strategies.

## Background

Demographic transition is accompanied by societal and political changes, such as an ageing labour force, the increase of the statutory retirement age (from 65 to 67 years, §35 BGB) and a prolonged working life for older employees. In 2019, the average age on reaching retirement was 64.1 years [1–3]. The objective of medical rehabilitation is to prevent early retirement by improving physical and mental health. Rehabilitation aims to create conditions for an independent life, minimising the limitations caused by chronic disease and to ensure participation in society and the labour market. In Germany, the German statutory pension insurance scheme is the main provider for rehabilitative care. Other providers are the statutory accident insurance (in the event of occupational accidents) and health insurances. These providers also cover the costs for rehabilitation measures. In Germany, an insured person can access medical rehabilitation after being referred by their physician [4–6]. Rehabilitation measures generally last around 3 weeks and are mainly provided in dedicated hospitals. In 2018, the German statutory pension insurance provided about 1.03 million rehabilitation measures. The average age of rehabilitation users was 53.5 years for women and 53.4 years for men [7].

The number of older employees with a migrant background (PMB) in the German workforce is increasing. PMB are born outside or in Germany, with at least one parent born abroad, or of foreign nationality [8]. Apart from the German Federal Statistical Office definition, studies often use other indicators to describe migrant status. This can be nationality, ethnic affiliation and mother tongue, making it difficult to compare findings [9]. In Germany, about 43 million people participate in the labour force, of which approximately 25% are PMB [8]. By 2020, an estimated two million PMB will be aged 60 years and older [10, 11]. The proportion of older employees is expected to grow faster among PMB than in the autochthonous (non-migrant) German population [12]. Ethnic-German resettlers (12.7%) from Eastern Europe and the former Soviet Union and persons with a Turkish (13.3%) and Polish (10.8%) migrant background account for the largest proportion of PMB [8].

Studies have shown that older employees with a migrant background, especially those of foreign nationality, have a higher risk of poorer occupational health than non-PMB in manual and low-skilled jobs. Incapacity to work and occupational diseases, or accidents are more common for foreign nationals. As are disability pensions, due to years of physically and mentally demanding occupations [13–16]. Less favourable health outcomes can be explained further by social inequalities (e.g. low socioeconomic status) in the destination country and by negative exposures (such as bad sanitary conditions or poorer job quality) in the country of origin, in Germany, or both [17]. Information deficits, due to insufficient German language skills, lack of knowledge of the health care system and cultural misunderstandings, result in PMB experiencing access barriers to health care. This leads to a lower utilisation, as well as to a limited effectiveness of health system services for foreign nationals, in comparison to German nationals, e.g. in rehabilitation services [13, 10, 17–21].

This scoping review assesses the utilisation of medical rehabilitation services by PMB (including foreign nationals) comparing groups of different origins, as well as PMB and non-PMB.

## Methods

We conducted a scoping review, which is a type of review that aims to identify the state of research, examines research gaps and gives a broad overview of a topic [22–25]. Our research question was specified with the **P**opulation-**I**ntervention-**C**omparison-**O**utcome criteria, which is used to formulate a research question when searching for relevant studies and evidence [26]: “Are there any differences in the utilisation of medical rehabilitation measures between PMB (and between subgroups of PMB) and non-PMB?”

### Inclusion and exclusion criteria

We searched for all study types—cohort studies, surveys, randomised clinical trials and qualitative studies—with a sample of persons of working age, 18 to 60+ years (**P**opulation), who have utilised medical rehabilitation services in Germany (**I**ntervention). We included studies which examined PMB compared to non-PMB, German nationals compared to foreign nationals and also studies comparing migrant subgroups (**C**omparison). We chose the following broad concept of utilisation as we expected few studies on the subject, specifically for the outcomes we have focussed on (**O**utcome): utilisation, medical indications, treatment outcomes and rehabilitation (treatment) success, work-related performance and returning to work after rehabilitation, satisfaction with rehabilitation, perceptions/expectations, as well as needs/intentions and barriers.

We excluded studies which did not fit our inclusion criteria and which did not consider the German rehabilitation system. We also included studies published from 2001 (after a change of the legislation covering rehabilitation), until the end of January 2020. Our search was conducted in 2018 and updated in 2020.

### Search matrix

**Fig. 1:**
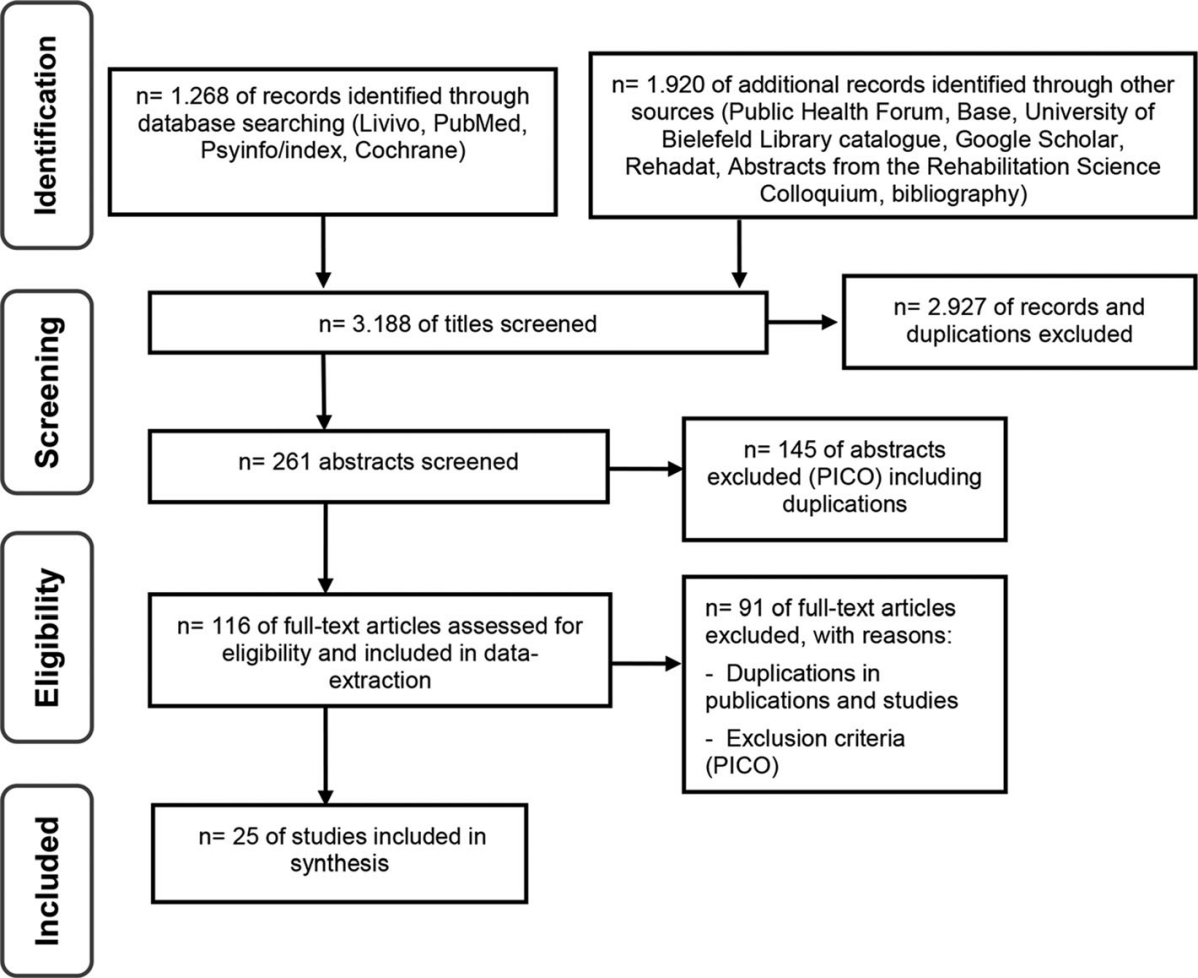
Scoping review search results

We carried out the search (see search terms in Table 1) in the following databases: Cochrane, Prospero, PubMed, Livivo and Psycindex/info. Due to only a small number of results, we also searched grey literature, including conference abstracts and project reports. Grey literature was identified in the “Base” and “Rehadat” databases, as well as in the Bielefeld University Library catalogue, in the non-indexed journal “Public Health Forum” and in the conference proceedings of the Rehabilitation Science Colloquium of the German Pension Insurance (DRV). For further information, we used a snowball system including literature by manual search from bibliography of the included studies and checked the citations in Google Scholar®. Some authors have been contacted to identify additional studies from grey literature, or to clarify questions regarding study design (Fig. 1).

**Table 1:** Main search: search terms and databases

Databases and grey literature*	Search term 1	Search term 2	Search term 3	Search term 4
	AND combinations of search words			
Cochrane, PROSPERO, Bielefeld University Library catalogue, Base, Public Health Forum, Psyindex/info	rehabilitation			
	rehabilitation	migra*		
	rehabilitation	migration(*)	migrant	migration(*)
	rehabilitation	migra*	German	
Rehadat	Migration			
Conference abstracts of the Rehabilitation Science Colloquium of the German Pension Insurance (DRV)	Migra*			
Livivo	rehabilitation	migration	Germany	
	rehabilitation	migrants		
	AND and OR combinations of search words			
PubMed	((tertiary prevention[MeSH] OR rehabilitation[MeSH Terms]) OR recovery) AND ((human migration[MeSH Terms] OR (immigrants and emigrants) OR non-German OR migration background[Text Word] OR transients and migrants[Mesh] OR foreign[Text Word])) AND Germany[MeSH]			

*Google Scholar and manual research has been used with the snowball system to identify publications from abstracts and to identify studies from bibliography in articles

For each database and source of grey literature, a different search approach was applied in order to broaden our search results. We mainly used search term combinations of “rehabilitation” AND “migra*”. In PubMed, we used a search term combination with the Boolean Operators AND as well as OR (Table 1).

Two reviewers independently searched and analysed the literature. We identified relevant titles, excluded duplicates and applied the inclusion and exclusion criteria to the titles. We then reviewed the abstracts. An additional third reviewer assessed studies when two reviewers differed in opinion. Some publications were only available as abstracts for the most important rehabilitation congress in Germany, the DRV Rehabilitation Science Colloquium and not published in (peer-reviewed) journals. We included the abstracts in our data extraction sheet and extracted the full text when available.

### Categories for the synthesis of the studies

We summarised the most relevant characteristics answering our research question in Tables 2 and 3. Here, we used the terms, concepts and definitions reported in the studies in the different sections, e.g. for the migrant background (see Table 3).

**Table 2:** Summary of technical information about the included studies

No.	Authors	Linked Source	Publication Type				Study design						Methods						
			Conference abstract	Original research paper	Report	Other (e.g. Book Chapter, documentation)	Cross-sectional	Longitudinal	Survey	Evaluation (Pre-Post)	Cohort	Qualitative	Primary data	Secondary data	data analysis methods^a^	Focus groups	Structured interviews	Semi-structured interviews	Expert interviews
1..	Aksakal et al. 2018 [27]	^b^	x					x		x		x	x		i,d,c		x		x
2.	Brause et al. 2010 [28]	[29, 30]			x		x					x		x	d,i,c	x		x	x
3.	Brzoska et al. 2019 [31]	[32]		x			x	x	x		x			x	i,d				
4.	Brzoska et al. 2019 [33]			x				x	x		x		x	x	i,d				
5.	Brzoska et al. 2017 [34]			x			x		x					x	i,d				
6.	Brzoska et al. 2016 [35]			x			x		x					x	i,d				
7.	Brzoska et al. 2012 [36]	[13]		x			x							x	i,d				
8.	Brzoska et al. 2010 [13]	[15, 37, 21, 38–40, 20]			x		x	x	x			x		x*	d,i,c	x	x	x	x
9.	Erbstößer/ Zollmann 2015 [41]			x			x							x	d				
10.	Göbber et al. 2010 [42]	[43]		x			x		x				x		d,i				
11.	Gruner et al. 2012 [44]			x				x			x		x		d,i				
12.	Höhne 2007a [45]					x	x							x	d				
13.	Höhne / Schubert 2007b [46]					x	x							x	d,i				
14.	Höhne et al. 2007c [47]		x				x							x	d,i				
15.	Jankowiak et al. 2018 [48]		x					x						x	d,i				
16.	Kaluscha et al. 2011 [49]		x					x			x			x	i				
17.	Kessemaier et al. 2019 [50]			x			x							x	d,i				
18.	MHH/ EMZ e.V. 2017 [51]	[52, 53, 19]			x			x		x		x	x		d,i,c	x	x		x
19.	Kohler/ Ziese 2004 [54]				x		x		x				x		d				
20.	Maier 2008 [55]			x				x						x	d				
21.	Pfeiffer et al. 2010 [43]	[42]				x	x		x				x		d				
22.	Ritter et al. 2017 [56]			x			x		x					x	d,i				
23.	Schröder et al. 2020 [57]			x				x			x		x		d,i				
24.	Yilmaz-Aslan et al. 2017 [58]	[59]	x				x					x			c	x		x	
25.	Zollmann et al. 2016 [60]			x			x							x	d				

^a^*d* descriptive analysis, *i* inductive analysis, *c* content analysis

^b^Personal Communication with Aksakal and Yilmaz-Aslan (March 2020)

**Table 3:** Summary of content-related information

No.	Authors	Linked source	Groups (and their origin) with and without migrant background							Differentiation of the migrant status						Research focus							Differences between groups
			Germans	Turkish	Former Yugoslavian	Former citizens of the Soviet-Union	Resettler	Mediterranean (S, G, P, I)	Other (e.g. non-Turkish, non-German, EU-nationals)	Nationality	Place of birth	Migration experience/status/ immigration after 1949	Spoken/native language	Other (e.g. PMB and non-PMB not specified)	Medical indications^a^	Utilisation of medical rehabilitation services	Barriers to medical rehabilitation services	Satisfaction with the utilisation of rehabilitation services	Perceptions/ expectations and needs/ intentions	Rehabilitation (treatment) success/ treatment outcome	Work-related performance	Return to work	
1.	Aksakal et al. 2018 [27]	^b^							o					x				x	x				Insufficient knowledge, treatment desires, language barriers for PMB compared to non-PMB
2.	Brause et al. 2010 [28]	[29, 30]		x					x	x^*^				x	1-6	x	x		x	x	x		Turkish PMB: lower rehabilitation success and work ability for musculoskeletal/connective tissue, mental illnesses metabolism/digestion and other (respiratory diseases) than for non-PMB Barriers for Turkish PMB (access, knowledge, language, culture)
3.	Brzoska et al. 2019 [31]	[32]	x						x	x	x		x	x		x							German nationals (non-PMB and PMB) and non-German nationals did not differ in their utilisation of rehabilitation
4.	Brzoska et al. 2019 [33]		x				x		x	x				X^c^		x							Foreigners (PMB) compared to Germans without Resettler status had a lower chance to use medical rehabilitation Resettler had a higher chance of using rehabilitation compared to foreigners
5.	Brzoska et al. 2017 [34]		x	x	x			x	x	x					1-3,6			x					Lower probability for satisfaction with rehabilitation for Turkish nationals (PMB) Other foreign nationals were as satisfied as German nationals (non-PMB)
6.	Brzoska et al. 2016 [35]		x	x	x			x	x	x										x	x		Non-Germans report less favourable outcomes, Turkish and former Yugoslavian origin have a higher chance for a poor treatment outcome than patients from Mediterranean countries
7.	Brzoska et al. 2012 [36]	[13]	x	x	x			x	x		x		x							x	x		Non-Germans showed a higher chance for low occupational performance after completing the rehabilitation a lower effectiveness of rehabilitation for Turkish and former Yugoslavian compared to Germans
8.	Brzoska et al. 2010 [13]	[15, 37, 21, 38–40, 20]	x	x	x		x	x	x	x	x		x	x	1-6	x	x		x	x	X		Non-Germans less utilised rehabilitation than Germans, Foreigner have a higher chance than Germans for occupational diseases, lower rehabilitation occupational performance and effectiveness after rehabilitation, PMB had barriers for utilisation compared to non-PMB: expectations, information deficit, missing intention to apply, language and culture
9.	Erbstößer/ Zollmann 2015 [41]		x	x	x	x		x	x	x				x	1-6	x				x	x	x	Non-Germans less utilised rehabilitation (heterogeneous for nationalities), had less often a full work performance after rehabilitation than Germans, Differences in indications e.g. for musculoskeletal rehabilitation: patients from former Soviet-Union utilised more than other nationals and Germans
10.	Göbber et al. 2010 [42]	[43]		x					x	x			x	x	2-5				x	x	x	x	PMB were pension-oriented, desired for gender-specific treatment PMB had a shorter treatment-duration, negative work performance (subjective), more mental and somatoform illnesses than non-PMB
11.	Gruner et al. 2012 [44]		x	x	x	x		x				x		x	5				x	x	x		PMB had a higher frequency for diseases, less work performance before and after rehabilitation (subjective), pension desire, are sicker at the beginning of rehabilitation, men (non-PMB) benefitted less
12.	Höhne 2007a [45]		x	x						X				x	1-3,5-6	x					x		Disabled pensioners (PMB) utilised less rehabilitation services in the last five years before retirement
13.	Höhne / Schubert 2007b [46]		x	x					X	x					1-6	x					x		Differences in the medical rehabilitation benefits between Germans and Non-Germans in the last 5 years before retirement: Non-German had a higher incapacity for work, mental illnesses affect non-Germans more than Germans
14.	Höhne et al. 2007c [47]		x						X	x					1-3,5-6	x				x	x		Differences in the utilisation of rehabilitation: Germans utilised more services of rehabilitation than PMB
15.	Jankowiak et al. 2018 [48]		x						x	x				x		x							Medical rehabilitation was less utilised by non-Germans than Germans, Applications for rehabilitation were lower for non-Germans than Germans
16.	Kaluscha et al. 2011 [49]		x	x	x			x	x	x					1-5	x							Turkish nationals utilise rehabilitation more often due to mental illnesses than Germans
17.	Kessemaier et al. 2019 [50]								x	x	x		x	x				x					PMB showed severe symptoms and were not as satisfied with the rehabilitation as non-PMB
18.	MHH/ EMZ e.V. 2017 [51]	[52, 53, 19]	x	x		x			x			x		x			x		x				Barriers to utilise rehabilitation: migrant specific (information deficit), person-related barriers (language), systematic-related barriers (bureaucracy), barriers independent of migrant status (fear of job loss), Increased application intention through the campaign for PMB
19.	Kohler/ Ziese 2004 [54]		x						x	x	x	x				x							PMB utilised rehabilitation less often than Germans
20.	Maier 2008 [55]			x					x	x				x	1-3,5	x				x			Turkish rehabilitants utilised more rehabilitation services than the non-Turkish, health did not improve as much as in non-Turkish, differences in indications: more musculoskeletal and mental illnesses for Turkish PMB
21.	Pfeiffer et al. 2010 [43]	[42]	x	x	x	x	x	x	x	x		x		x	2-4				x	x		x	PMB were pension-oriented, had a shorter treatment-duration, more mental and somatoform illnesses than non-PMB, other treatment expectations than non-PMB
22.	Ritter et al. 2017 [56]		x						X	x					2	x							Lower chance of utilisation for foreign nationals with hip and knee arthroplasty compared to Germans
23.	Schröder et al. 2020 [57]		x						x	x	x	x				x							First-generation migrants had a lower chance of utilising outpatient rehabilitation than non-migrants No differences between first- and second-generation migrants and non-migrants for any rehabilitation
24.	Yilmaz-Aslan et al. 2017 [58]	[59]	x	x									x	x	1	x	x	x	x				Turkish PMB in comparison to Germans: higher need for support (information and emotional), psycho-oncological care was rarely utilised, barriers for Turkish-PMB e.g. information deficit, culture, language and prejudice
25.	Zollmann et al. 2016 [60]		x	x	x				x	x					5	x				x	x	x	Turkish PMB is largest group in psychosomatic rehabilitation (year 2012), were sicker at the beginning of rehabilitation than non-Turkish nationals, reintegration into working life is less successful for them

^a^*1* neoplasms, *2* muscle/skeletal/connective tissue, *3* cardiovascular, *4* metabolism/digestion, *5* mental illnesses (incl. addiction), *6* other (not described, e.g. respiratory diseases)

^b^Personal Communication with Aksakal and Yilmaz-Aslan (March 2020)

^c^identification of resettlers from information about prior occupation in the country of origin in routine data

*application of a name-based algorithm to identify Turkish rehabilitants in the data from the German statutory pension insurance

Table 2 provides an overview of the technical characteristics of the selected studies, e.g. publication type, study design and methods. Only the most frequent characteristics are depicted in subcategories (Tables 2 and 3). Table 3 summarises content-related information of the studies and helps in answering our research question, e.g. which PMB groups were studied, how migrant status was defined, and main results*.* In both tables, the category “linked source” is integrated to show that some studies, or parts of studies are published and described in shorter or more detailed form, e.g. in other journals or books. We explained and included more detailed results for interested readers in the full text and in our additional files. We showed the most frequently used outcomes of the studies in the category “research focus” (Table 3) and grouped outcomes, e.g. rehabilitation (treatment) success with treatment outcome, because of their related content.

From 3188 identified sources, we selected 261, after removal of duplicates and using the inclusion/exclusion criteria on the titles. After reviewing the abstracts, another 145 could be excluded according to PICO-criteria including duplicates. We screened and extracted 116 full texts. After identifying further duplicates and applying the inclusion/exclusion criteria on the full texts, 25 studies were available for the synthesis and analysis (Fig. 1).

## Results

### Characteristics of the studies

About half of the included studies were published as original research papers (13 of 25). Five studies were published in conference abstract books and four as final project reports. Lastly, we identified three studies through other sources, such as documentation, presentation at a conference or book chapters (Table 2 and Additional file 1: Table S1). Of 25 studies, 17 applied secondary data analyses. Of the 17 secondary data analyses, eleven used a cross-sectional approach, four a longitudinal approach and two mixed cross-sectional and longitudinal elements. Two of the secondary data analyses were part of mixed method approaches including qualitative sub-studies (Table 2). Additionally, we identified two mixed-method approaches using focus groups and semi-structured interviews, or structured interviews combined with an evaluation. Five qualitative (sub-) studies used focus groups (4), semi-structured interviews (4), structured interviews (3) or expert interviews (3).

In total, only one of the twenty-five studies followed a purely qualitative approach. Fourteen studies examined routine data from the German statutory pension insurance at a state and federal level (see Additional file 1: Table S1): data from a quality assurance survey, routine data and routine data from the German statutory pension insurance, combined with data from a German statutory health insurance, the German employment agency, the German statutory accident insurance and the German Socio-Economic Panel [12]. Five studies used primary data from quantitative surveys. One study analysed data from the Socio-Medical Panel [30]. Another cohort study analysed data routinely collected from two rehabilitation hospitals. In most cases, quantitative studies applied inductive and descriptive statistics (16) or only descriptive statistics (5). Content analysis (5) was the method of choice for qualitative approaches. More detailed information is depicted in Additional file 1: Table S2.

Only one study included all major migrant groups in their analysis (see Table 3). Seven studies focused on differences between persons from Germany, Turkey, former Yugoslavia and Mediterranean countries (Portugal, Spain, Italy and Greece), through the indicator “nationality”. Three surveys examined resettlers: a qualitative sub-study, a secondary data analysis and a survey. Two other studies included citizens from the former Soviet Union in the groups compared (Table 3). Another study focused specifically on Germans, resettlers and foreign nationals. Four studies compared results by “nationality” only, one defined PMB by “native language” and “years lived in Germany” and another three differentiated PMB and non-PMB through “place of birth”, “nationality” and “migrant status/experience”, or “native language” (Table 3). The last of the two studies differentiated the migrant status with the categories: “one/two-sided migrant background” and ”migration status (yes/no)”, using “place of birth (of the parents)” and “nationality”.

The most frequently used definition for the migrant status was “nationality”—a consequence of the high number of publications using routine data (16 of 25 studies), e.g. of the German statutory pension insurance. Fewer studies analysed “place of birth” of the persons and/or of their parents (8 of 25) and “(native/spoken) language” (6 of 25), using a combination between “place of birth” and “(native/spoken) language” in three cases. In addition, two studies applied a name-based algorithm to identify Turkish rehabilitants in the data from the German statutory pension insurance (Additional file 1: Table S2) [27, 28], or identified resettlers from information about prior occupation in the country of origin in routine data (1) [33].

### Main results

The most frequently examined research foci in the studies were “utilisation of rehabilitation services” (16 of 25 studies) and the “work-related performance” (12 of 25). Ten of 25 studied the aspects “rehabilitation (treatment) success/treatment outcome” after utilisation of rehabilitation (Table 3). Also, 5 of 25 studies analysed “barriers”, e.g. access or effectiveness of rehabilitation, and eight studies analysed “perceptions/expectations”, as well as “needs/intentions”. Fifteen studies researched diverse “indications” for rehabilitation between the migrant groups (Additional file 1: Table S2). Other aspects examined in studies were differences in “satisfaction“ of rehabilitants with the utilisation of rehabilitation (4) (Table 3) followed by the ability to “return to work“ after completing the rehabilitation (4 of 25). In nine cases, studies examined the outcomes, “rehabilitation (treatment) success/treatment outcome” and “(work-related) performance”, applying secondary data analysis (7). Five of them were based on routine data and less on survey data (2). Nine studies combined “medical indications” with “rehabilitation (treatment) success/treatment outcome” and “(work-related) performance”. The aspect “perceptions/expectations/needs/intentions” was linked to “barriers” four times (Table 3) and in half of the cases combined through qualitative studies and sub-studies. Two mixed-method studies linked the “rehabilitation (treatment) success/treatment outcome”, “(work-related) performance”, the ability to “return to work” with the aspect of “barriers” in mixed-method studies.

About half of the studies examined differences between PMB and non-PMB with a focus on the indications muscle/skeletal/connective tissue (12) and mental illnesses (12). This was followed by neoplasms (11) and diseases of the cardiovascular system (11). Indications relating to metabolism and digestion were investigated less frequently (6). Four studies analysed all of the aforementioned indications (Additional file 1: Table S2). Of 25 studies, 15 investigated a combination of inpatient and outpatient care (Additional file 1: Table S2). Inpatient care was examined in eight studies, whereas outpatient care was only assessed once. Studies focused mostly on data of rehabilitants obtained after rehabilitation was completed (20) (Additional file 1: Table S1).

“Differences in the utilisation of medical rehabilitation services” are summarised in Table 3 and in the following text. The diverse groups of PMB were heterogeneous in their utilisation of medical rehabilitation [36, 39, 41–43]. Non-German nationals used medical rehabilitation services less frequently than those with German nationality [13, 32, 33, 45, 46, 48, 56, 54, 47, 31]. Jankowiak et al. (2018) [48], Brzoska et al. [33, 35, 36, 39] and other authors [45, 47, 56] identified a significantly lower chance for foreign nationals to utilise medical rehabilitation in comparison to German nationals. Kohler and Ziese (2004) presented similar results in more detail for PMB and Germans (non-PMB) in a representative survey [54]. In recent representative studies, e.g. the Socio-Medical Panel [29] or the lidA-cohort study [57], where the migrant background was examined in detail, no significant differences between PMB and non-PMB were found for rehabilitation in general.

In comparison to non-PMB, PMB and foreign nationals faced more barriers in access to care (e.g. unaddressed cultural and religious needs) and a lower effectiveness of rehabilitation due to a lack of culture- or gender-sensitive treatment concepts of health care providers [13, 27, 28, 35, 51, 42]. Moreover, discrimination and inadequate communication have been identified for PMB [13, 19, 59, 21, 27, 28, 34, 50]. This is, for example, due to language barriers, illiteracy or communicating symptoms differently. PMB had more need for (psychological) support and expected better results from treatment and rehabilitation than non-PMB did. This was identified for Turkish PMB [13, 34, 50, 59, 58] in particular. Kessemeier et al. (2019) found in their cohort study that PMB were less satisfied with rehabilitation services [50]. In particular, Turkish nationals Brzoska et al. (2010) showed less satisfaction with treatment and care in rehabilitation than other PMB, e.g. foreign nationals from the former Yugoslavia and Mediterranean countries. The last two groups were just as satisfied as the German rehabilitants [13]. Beyond the access barriers, PMB showed a lower intention to [51, 59] apply and use medical rehabilitation [13, 48, 51]. Another study, which differentiated only between Germans and non-Germans, showed that non-Germans had less improved health or treatment outcome even after considering the confounders, such as education, socio-economic status and health [35, 41].

The included studies showed that effectiveness of rehabilitation and rehabilitation success is lower for PMB than for non-PMB. This included a lower (work-related) performance and less capability to return to work [28, 35, 36, 41, 44, 55, 60, 42]. Further, PMB considered their own work performance more negative than non-PMB [34, 35, 44]. In comparison to PMB, non-Germans had a lower ability to work after rehabilitation, but they had a higher probability of receiving disability pensions. Their reintegration into working-life was less successful than for German nationals [41, 45, 46, 60, 39]. Many studies identified that PMB had a lower level of education and vocational training and often work in more physically and demanding jobs than non-PMB [50, 57, 13, 35, 29, 44, 47, 60, 42]. For non-Germans, we identified a shorter work duration, higher rates of occupational disease and accident pension [13, 41, 46, 15]. The results generally included the PMB sub-group of foreign nationals [13, 28, 29, 34, 45, 46].

Additionally, Erbstößer and Zollmann (2015) [41] reported longer durations of inability to work 12 months before rehabilitation for Turkish nationals than for German nationals. In contrast, rehabilitants from the former Soviet Union had a shorter duration of inability to work than Germans did. While Erbstößer and Zollmann (2015) [41] did not find any differences for non-German nationals in treatment duration, Pfeiffer et al. (2010) [43] and Göbber et al. (2010) [42] found a shorter treatment duration for Turkish rehabilitants with migrant background. Turkish rehabilitants with migrant background, as well as Turkish nationals used rehabilitation services more frequently than non-Turkish rehabilitants [28, 55, 60, 42] and were younger than non-Turkish rehabilitants [29, 28, 39].

Important differences between German and non-German nationals, as well as between different PMB groups were seen in secondary data analyses in terms of indications. PMB used more rehabilitation services because of indications relative to “muscle/skeletal/connective tissue”, “mental illness (incl. addiction)” and “neoplasms” [41, 60]. However, there were hardly any differences for “cardiovascular” indications. In comparison, rehabilitants with Turkish origin utilised more “muscle/skeletal/connective tissue” and “mental health”-related rehabilitations than other PMB and non-PMB [28, 29, 49, 55, 60] (Additional file 1: Table S2)*.*

## Discussion

We identified 25 studies and found that non-German nationals used medical rehabilitation services less frequently than persons with German nationality. Moreover, we discovered differences in several aspects of the utilisation of rehabilitation services between PMB and non-PMB and between different migrant groups. These differences were statistically non-significant or did not have much of an effect on the use of rehabilitation services [57, 31]. PMB and foreign nationals showed a less favourable health status, had a lower education level and had a higher prevalence of occupational diseases [14, 40, 42, 47, 60, 16, 51]. These characteristics may have influenced the outcome of rehabilitation services between PMB and non-PMB additionally.

The use of services was mainly investigated for the group of Turkish nationals and PMB in general, followed by nationals from former Yugoslavia, the former Soviet Union and Mediterranean countries. Fewer studies and sub-studies examined ethnic-German resettlers or focused on other migrant groups, e.g. Polish PMB, or PMB of Arabic origin living in Germany. Among the databases, we found five qualitative studies examining the use of rehabilitation and other aspects. In recent studies (published since 2018), no clear evidence for differences in utilisation of PMB in comparison to non-PMB was found. This could indicate a change in rehabilitation utilisation over time. Possible reasons could be the changes in service provision towards a more diversity-sensitive approach. Besides, young PMB may be more knowledgeable and willing to use health services such as medical rehabilitation [61, 62]. Another aspect could be that the PMB are better informed about the possibilities of using medical rehabilitation or that recent studies do record information about migrant background more precisely [57, 31].

In comparison to our results, other countries showed a lower use of medical rehabilitation for ethnic minorities. In the USA, non-Whites (for example Blacks and Hispanics) had a lower chance of discharge to rehabilitation in post-hospitalisation care after trauma than Whites, thereby not having the chance to use rehabilitation [63]. In a Canadian register-based study on cardiac rehabilitation programs, similar results were presented for ethno-cultural minorities compared to white patients. In a review, Ellis and Egede (2014) found [64], when comparing racial differences in post-stroke rehabilitation in the USA, ambiguous results based on four studies. Nevertheless, they suspected the existence of racial differences in their review.

There are limitations comparing the utilisation of specific health care services, such as rehabilitation, between Germany and other countries. The rehabilitation systems vary widely due to their historical development. While in Germany medical rehabilitation takes place in hospitals away from the place of residence (mainly as inpatient care), the opposite is the case in other European countries [36, 65, 66]. In this review, most studies did not explicitly differentiate between the use of outpatient (at the place of residence) and inpatient rehabilitation.

Further possible causes for our results could be that there are other influencing factors for the utilisation of health services such as medical rehabilitation, e.g. social inequalities. Socioeconomic-related inequalities can influence the utilisation of health services worldwide [67–69]. In regard to rehabilitation, Jankowiak et al. (2018) found that persons with a higher social status did use medical rehabilitation more often than those with a lower social status [48].

We identified barriers such as lack of information, knowledge of the rehabilitation system or cultural barriers. Also, unaddressed needs, as well as dissatisfaction can hinder utilisation and success of rehabilitation [28, 27, 70]. Enabling factors like resources, intentions or motivations, as well as diversity aspects were rarely investigated.

Nationality is the common characteristic for migrant background registered in German routine data [71]. Insufficient differentiation in routine data of persons with a migrant background is evident throughout Europe [66]. There is not yet a routine data basis that allows to differentiate between PMB of German and foreign nationality or to compare persons with and without a migrant background by recorded migration characteristics [71, 66]. However, there are also positive examples, such as the Netherlands and Finland, which record information on the country of birth in addition to nationality. Furthermore, routine data in the Netherlands records the countries of birth of the parents [66].

### Strengths and limitations

We included studies indexed in databases and grey literature that we could identify online. The literature search was not as comprehensive as in a systematic review. Our aim was to examine the state of research, research gaps and to give an overview on the current state of knowledge on the research focus. Our search resulted in the inclusion of 25 studies which focussed on the use of medical rehabilitation services. A possible reason for the low number of included studies is the difficulty of recruiting PMB for studies [72]. Besides, in Germany only a small number of researchers conduct research in the context of migrants/foreigners and use of rehabilitation. Moreover, the included migrant groups are heterogeneous and less than half of the studies and sub-studies had the opportunity to examine indicators other than nationality to differentiate the migrant status. Consequently, not all persons with migrant background were included, but principally those with a German nationality were missing. Therefore, we assume a lack of representativeness of the present results. It is not possible to conclude that there is a lower utilisation of medical rehabilitation through PMB in comparison to non-PMB, as well as that there are barriers for utilisation. Most of available research results can only explain a lower utilisation for foreign nationals. To prevent their early retirement, access and effectiveness barriers [39, 19] before and during utilisation of rehabilitation need to be reduced. In addition, the rehabilitation success, improvement of health outcomes, the effectiveness of rehabilitation and possible resources for utilisation among PMB should be examined. One new approach to improve rehabilitation utilisation for PMB is diversity-sensible strategies in hospitals and rehabilitation hospitals, but their effectiveness remains to be evaluated [70].

## Conclusion

We did not find differences in the utilisation of rehabilitation services in recent studies between PMB and non-PMB. However, we found out that in previous studies, non-German nationals used medical rehabilitation services less frequently than persons with German nationality. There is a need for studies examining the use of rehabilitation and assessing migrant background by more than just nationality to allow more precise comparisons between PMB and non-PMB. Comparative studies, which survey more than one aspect of utilisation, are also important in order to detect possible differences between groups. Furthermore, there is a need for research focusing on factors enabling utilisation of rehabilitation, improving health outcomes after rehabilitation and evaluating diversity-sensible strategies in order to understand possible changes in the provision of rehabilitation services.

## Supplementary information

**Additional file 1: Table S1**. Studies, technical information to answer the review question. **Table S2**. Studies, content-related information to answer the review question

## Data Availability

Not applicable

## Abbreviations

non-PMB: Persons without a migrant background
PMB: Persons with a migrant background

## References

[1] Hasselhorn HM, Ebener M, Müller BH. Determinanten der Erwerbsteilhabe im höheren Erwerbsalter – das “lidA-Denkmodell zu Arbeit, Alter und Erwerbsteilhabe”. Z Sozialreform 2015; 61(4). 10.1515/zsr-2015-0404.

[2] Walter N, Fischer H, Hausmann P, Klös H-P, Lobinger T, Raffelhüschen B et al. Die Zukunft der Arbeitswelt. Auf dem Weg ins Jahr 2030; 2013 [cited 2020 Mar 1]. Available from: https://www.bosch-stiftung.de/sites/default/files/publications/pdf_import/Studie_Zukunft_der_Arbeitswelt_Einzelseiten.pdf. Accessed 1 March 2020.

[3] Deutsche Rentenversicherung Bund. Rentenversicherung in Zahlen 2019. Statistik der Deutschen Rentenversicherung. Berlin; 2019.

[4] Viehmeier S, Schubert M, Thimmel R. Vor der Rehabilitation. In: Rehabilitation: Vom Antrag bis zur Nachsorge - für Ärzte, Psychologische Psychotherapeuten und andere Gesundheitsberufe. Berlin: Springer; 2018. p. 181–95 [Springer Reference Medizin]. 10.1007/978-3-662-54250-7_18.

[5] Thimmel R, Schubert M, Viehmeier S. Übergreifende Aspekte zum Reha-Prozess. In: Rehabilitation: Vom Antrag bis zur Nachsorge - für Ärzte, Psychologische Psychotherapeuten und andere Gesundheitsberufe. Berlin: Springer; 2018. p. 217–28 [Springer Reference Medizin]. 10.1007/978-3-662-54250-7_21.

[6] Stucki G, Bickenbach J, Gutenbrunner C, Melvin J (2018). Rehabilitation: the health strategy of the 21st century. J Rehabil Med.

[7] Deutsche Rentenversicherung Bund. Die medizinische und berufliche Rehabilitation der Rentenversicherung im Licht der Statistik mit dem Fokusthema “Gestärkt ins Leben: Kinder- und Jugendrehabilitation der Rentenversicherung”. Berlin; 2020.

[8] Statistisches Bundesamt (Destatis). Bevölkerung mit Migrationshintergrund: - Ergebnisse des Mikrozensus 2018 -; Bevölkerung und Erwerbstätigkeit. Fachserie 1 Reihe 2.2 [cited 2020 Mar 1]. Available from: https://www.destatis.de/DE/Themen/Gesellschaft-Umwelt/Bevoelkerung/Migration-Integration/Publikationen/Downloads-Migration/migrationshintergrund-2010220187004.pdf?__blob=publicationFile. Accessed 1 March 2020.

[9] Schenk L, Bau A-M, Borde T, Butler J, Lampert T, Neuhauser H (2006). Mindestindikatorensatz zur Erfassung des Migrationsstatus. Bundesgesundheitsbl Gesundheitsforsch Gesundheitsschutz.

[10] Razum O, Meesmann U, Bredehorst M, Brzoska P, Dercks T, Glodny S et al. Schwerpunktbericht: Migration und Gesundheit; 2008 [cited 2020 Mar 1]. Available from: https://edoc.rki.de/bitstream/handle/176904/3194/253bKE5YVJxo_28.pdf?sequence=1, https://edoc.rki.de/bitstream/handle/176904/3194/253bKE5YVJxo_28.pdf?sequence=1. Accessed 1 March 2020.

[11] Weber A, Hörmann G (2011). Migration und Gesundheit--von Defizitanalysen zum Diversity-Ansatz?. Gesundheitswesen.

[12] Burkert C, Hochfellner D, Wurdack A. Ältere Migrantinnen und Migranten am Arbeitsmarkt. In: Baykara-Krumme H, Motel-Klingebiel A, Schimany P, editors. Viele Welten des Alterns: Ältere Migranten im alternden Deutschland. Wiesbaden: Springer VS; 2012. p. 77–100 [Alter(n) und Gesellschaft; vol. 22]. 10.1007/978-3-531-19011-2_3 .

[13] Brzoska, P., Voigtländer, S., Reutin, B., Yilmaz-Aslan, Y., Barz, I., Starikow, K., et al. Rehabilitative Versorgung und gesundheitsbedingte Frühberentung von Personen mit Migrationshintergrund in Deutschland: Abschlussbericht; 2010 [cited 2020 Mar 1]. Available from: http://www.bmas.de/SharedDocs/Downloads/DE/PDF-Publikationen/forschungsbericht-f402-rehabilitation-migrationshintergrund.pdf?__blob=publicationFile. Accessed 1 March 2020.

[14] Oldenburg C, Siefer A, Beermann B. Migration als Prädiktor für Belastung und Beanspruchung? In: Badura B, Schröder H, Klose J, Macco K, editors. Vielfalt managen: Gesundheit fördern - Potenziale nutzen. Berlin, Heidelberg: Springer; 2010. p. 141–51 [Fehlzeiten-Report]. 10.1007/978-3-642-12898-1_14.

[15] Brzoska P, Voigtlander S, Spallek J, Razum O. Arbeitsunfälle, Berufskrankheiten und Erwerbsminderung bei Menschen mit Migrationshintergrund. In: Schott T, Razum O, editors. Migration und medizinische Rehabilitation. 1st ed.: Beltz Juventa Verlag; 2013. p. 49–61.

[16] Schimany P, Baykara-Krumme H. Zur Geschichte und demografischen Bedeutung älterer Migrantinnen und Migranten in Deutschland. In: Baykara-Krumme H, Motel-Klingebiel A, Schimany P, editors. Viele Welten des Alterns: Ältere Migranten im alternden Deutschland. Wiesbaden: Springer VS; 2012. p. 43–73 [Alter(n) und Gesellschaft; vol. 22]. 10.1007/978-3-531-19011-2_2.

[17] Spallek J, Zeeb H, Razum O (2011). What do we have to know from migrants′ past exposures to understand their health status? A life course approach. Emerg Themes Epidemiol.

[18] Brzoska P, Yilmaz-Aslan Y, Razum O, editors. Zugang und Wirksamkeit bei der medizinischen Rehabilitation für Menschen mit Migrationshintergrund: Public Health Forum 2011;18 (4). p. 21.e1-e3. 10.1016/j.phf.2011.10.003.

[19] Schwarz B, Markin K, Salman R, Gutenbrunner C (2015). Barriers for migrants regarding the access to medical rehabilitation on behalf of the German Pension Insurance. Rehabilitation (Stuttg).

[20] Yilmaz-Aslan Y, Brzoska P, Schott T, Razum O. Reha aus Sicht von türkischen Migrant(inn)en. In: Schott T, Razum O, editors. Migration und medizinische Rehabilitation. 1st ed.: Beltz Juventa Verlag; 2013. p. 162–94.

[21] Reiss K, Yilmaz-Aslan Y, Reutin B, Barz I, Starikow K, Schott T et al. Reha aus der Sicht von Aussiedler(inne)n. In: Schott T, Razum O, editors. Migration und medizinische Rehabilitation. 1st ed.: Beltz Juventa Verlag; 2013. p. 162–94.

[22] Anderson S, Allen P, Peckham S, Goodwin N (2008). Asking the right questions: scoping studies in the commissioning of research on the organisation and delivery of health services. Health research policy and systems.

[23] Davis K, Drey N, Gould D (2009). What are scoping studies? A review of the nursing literature. Int J Nurs Stud.

[24] Tricco AC, Lillie E, Zarin W, O’Brien KK, Colquhoun H, Levac D (2018). PRISMA Extension for Scoping Reviews (PRISMA-ScR): checklist and explanation. Ann Intern Med.

[25] Peters M, Godfrey C, McInerney P, Baldini Soares C., Khalil H, Parker D. Chapter 11: scoping reviews. In: Aromataris E, Munn Z, editors. Joanna Briggs Institute Reviewer's Manual.; 2017 [cited 2020 Mar 1]. Available from https://reviewersmanual.joannabriggs.org/. Accessed 1 March 2020.

[26] Richardson WS, Wilson MC, Nishikawa J, Hayward RS (1995). The well-built clinical question: a key to evidence-based decisions. ACP J Club.

[27] Aksakal, T., Yilmaz-Aslan, Y., Akbulut, N., Razum, O., Brzoska, P. Umsetzung einer diversitätssensiblen Versorgung in Rehabilitationseinrichtungen. Ergebnisse einer exemplarischen Ist-Analyse. In: Deutsche Rentenversicherung Bund, editor. 27. Rehabilitationswissenschaftliches Kolloquium Deutscher Kongress für Rehabilitationsforschung: Rehabilitation bewegt! vom 26. bis 28. Februar 2018 in München; Sonderausgabe der DRV. Berlin; 2018. p. 512–4 [DRV Schriften; vol. 113]. [cited 2020 Mar 6]. Available from: http://forschung.deutsche-rentenversicherung.de/ForschPortalWeb/ressource?key=tagungsband_27_reha_kolloqu.pdf. Accessed 6 March 2020.

[28] Brause M, Reutin B, Schott T, Yilmaz-Aslan Y. Migration und gesundheitliche Ungleichheit in der Rehabilitation: Versorgungsbedarf und subjektive Bedürfnisse türkischer und türkischstämmiger Migrant(inn)en im System der medizinischen Rehabilitation: -Abschlussbericht -; 2010 [cited 2020 Mar 3]. Available from https://www.uni-bielefeld.de/gesundhw/zfv/endbericht.pdf. Access 3 March 2020.

[29] Brause M, Reutin B, Razum O, Schott T (2012). Rehabilitation results of Turkish immigrants - an analysis of routine data from the Rhineland and Westfalia Pension Insurance. Rehabilitation (Stuttg).

[30] Brause M, Schott T. Reha-Inanspruchnahme und Erfolg bei Menschen mit türkischem Migrationshintergrund. In: Schott T, Razum O, editors. Migration und medizinische Rehabilitation. 1st ed.: Beltz Juventa Verlag; 2013. p.62–91.

[31] Brzoska P, Spanier K, Bethge M (2019). Potenziale des Dritten Sozialmedizinischen Panels für Erwerbspersonen (SPE-III) für die Forschung im Bereich Migration und Rehabilitation: Das Beispiel der Inanspruchnahme rehabilitativer Versorgung. Rehabilitation (Stuttg).

[32] Brzoska P. Inanspruchnahme rehabilitativer Versorgung bei Menschen mit Migrationshintergrund. Untersuchungspotenziale des “Dritten Sozialmedizinischen Panels für Erwerbspersonen” (SPE-III). In: Deutsche Rentenversicherung Bund, editor. 27. Rehabilitationswissenschaftliches Kolloqiuim Deutscher Kongress für Rehabilitationsforschung: Rehabilitation bewegt! vom 26. bis 28. Februar 2018 in München; Sonderausgabe. Berlin; 2018. p. 231–3 [DRV Schriften 113] [cited 2020 Mar 6]. Available from: http://forschung.deutsche-rentenversicherung.de/ForschPortalWeb/ressource?key=tagungsband_27_reha_kolloqu.pdf. Accessed 6 March 2020.

[33] Brzoska P, Razum O (2019). Inanspruchnahme medizinischer Rehabilitation im Vorfeld der Erwerbsminderungsrente: Vergleich ausländischer und deutscher Staatsangehöriger unter besonderer Berücksichtigung von (Spät-)Aussiedler/-innen. Z Gerontol Geriatr.

[34] Brzoska P, Sauzet O, Yilmaz-Aslan Y, Widera T, Razum O (2017). Satisfaction with rehabilitative health care services among German and non-German nationals residing in Germany: a cross-sectional study. BMJ Open.

[35] Brzoska P, Sauzet O, Yilmaz-Aslan Y, Widera T, Razum O (2016). Self-rated treatment outcomes in medical rehabilitation among German and non-German nationals residing in Germany: an exploratory cross-sectional study. BMC Health Serv Res.

[36] Brzoska P, Voigtlander S, Spallek J, Razum O (2012). Die Nutzung von Routinedaten in der rehabilitationswissenschaftlichen Versorgungsforschung bei Menschen mit Migrationshintergrund: Möglichkeiten und Grenzen: potential and limitations. Gesundheitswesen.

[37] Yilmaz-Aslan Y, Brzoska P, Schott T, Razum O. Reha aus Sicht von türkischen Migrant(inn)en. In: Schott T, Razum O, editors. Migration und medizinische Rehabilitation. 1st ed.: Beltz Juventa Verlag; 2013. p. 195–201.

[38] Brzoska P, Voigtlander S, Spallek J, Razum O. Reha-Erfolg bei Migrant(inn)en: Herkunftsländer im Vergleich. In: Schott T, Razum O, editors. Migration und medizinische Rehabilitation. 1st ed.: Beltz Juventa Verlag; 2013. p. 105–10.

[39] Brzoska P, Voigtlander S, Spallek J, Razum O (2010). Utilization and effectiveness of medical rehabilitation in foreign nationals residing in Germany. Eur J Epidemiol.

[40] Voigtländer S, Brzoska P, Spallek J, Exner AK, Razum O. Die Inanspruchnahme medizinischer Rehabilitation bei Menschen mit Migrationshintergrund. In: Schott T, Razum O, editors. Migration und medizinische Rehabilitation. 1st ed.: Beltz Juventa Verlag; 2013. p. 92-104.

[41] Erbstößer S, Zollmann P (2015). Versorgungsunterschiede zwischen deutschen und ausländischen Rehabilitanden. RVaktuell.

[42] Göbber J, Pfeiffer W, Winkler M, Kobelt A, Petermann F (2010). Stationäre psychosomatische Rehabilitationsbehandlung von Patienten mit türkischem Migrationshintergrund: Spezielle Herausforderungen und Ergebnisse der Behandlung. Z Psychiatr Psychol Psychother.

[43] Pfeiffer W, Göbber J, Winkler M, Kobelt A, Petermann F. Stationäre psychosomatische Rehabilitationsbehandlung von Versicherten mit Migrationshintergrund. Neue Ansätze in der psychosomatischen Rehabilitation. Regensburg: S. Roderer Verlag 2010:49–70.

[44] Gruner A, Oster J, Müller G, von Wietersheim J (2012). Symptomatik, Krankheitsmodelle, Behandlungserleben und Effekte bei Patienten mit und ohne Migrationshintergrund in der psychosomatischen Rehabilitation. Z Psychosom Med Psychother.

[45] Höhne A. Erwerbsminderungsrenten und medizinische Rehabilitation in Deutschland unter Berücksichtigung des Migrationshintergrunds: 12. bundesweiter Kongress Armut und Gesundheit. Berlin; 2006 Dec 1. Präventionen für gesunde Lebenswelten - “Soziales Kapital” als Investition in Gesundheit.

[46] Höhne A, Schubert M. Vom Healthy-migrant-Effekt zur gesundheitsbedingten Frühberentung. Erwerbsminderungsrenten bei Migranten in Deutschland. In: Deutsche Rentenversicherung Bund, editor. Etablierung und Weiterentwicklung. Bericht vom vierten Workshop des Forschungsdatenzentrums der Rentenversicherung (FDZ-RV) am 28. und 29. Juni 2007 im Wissenschaftszentrum Berlin für Sozialforschung (WZB). Berlin; 2007. p. 103–25 [DRV-Schriften; vol. 55] [cited 2020 Mar 6]. Available from: http://forschung.deutsche-rentenversicherung.de/FdzPortalWeb/getRessource.do?key=drv_band_55_s_103_126_hoene.schub.pdf. Accessed 6 March 2020.

[47] Höhne A, Behrens J, Schubert M, Schaepe C, Zimmermann M. Das Krankheitsspektrum von Erwerbsminderungsrentnern mit Migrationshintergrund. In: Deutsche Rentenversicherung Bund, editor. 16. Rehabilitationswissenschaftlichen Kolloquium: Gesund älter werden -mit Prävention und Rehabilitation vom 26. März bis 28. März 2007 in Berlin. Berlin; 2007. p. 207–9 [DRV-Schriften; vol. 72]. Available from: http://forschung.deutsche-rentenversicherung.de/ForschPortalWeb/ressource?key=tagungsband_16_reha-kolloqu.pdf. Accessed 6 March 2020.

[48] Jankowiak, S., Kaluscha, R., Krischak, G. Soziale Unterschiede bei der Beantragung und Inanspruchnahme von medizinischen und beruflichen Rehabilitationsleistungen. In: Deutsche Rentenversicherung Bund, editor. 27. Rehabilitationswissenschaftliches Kolloquium Deutscher Kongress für Rehabilitationsforschung: Rehabilitation bewegt! vom 26. bis 28. Februar 2018 in München. Berlin; 2018. p. 504–7 [DRV Schriften; vol. 113] [cited 2020 Mar 6]. Available from: http://forschung.deutsche-rentenversicherung.de/ForschPortalWeb/ressource?key=tagungsband_27_reha_kolloqu.pdf. Accessed 6 March 2020.

[49] Kaluscha R, Brzoska P, Jacobi E, Krischak G. Inanspruchnahme medizinischer Rehabilitation wegen psychischer Erkrankungen: Gibt es Unterschiede zwischen Menschen deutscher und ausländischer Staatsangehörigkeit? In: Rehabilitationswissenschaftliches Kolloquium: Nachhaltigkeit durch Vernetzung; 2011. p. 141–2 [DRV-Schriften; vol. 93] [cited 2020 Mar 6]. Available from: http://forschung.deutsche-rentenversicherung.de/ForschPortalWeb/ressource?key=tagungsband_20_reha_kolloqu.pdf. Accessed 6 March 2020.

[50] Kessemeier FM, Bassler M, Petermann F, Kobelt-Pönicke A (2019). Therapeutische Allianz und Rehabilitationszufriedenheit von Menschen mit Migrationshintergrund in der psychosomatischen Rehabilitation – Analyse routinemäßig erhobener Daten. Physikalische Medizin, Rehabilitationsmedizin, Kurortmedizin.

[51] Klinik für Rehabilitationsmedizin, Medizinische Hochschule Hannover (MHH), Ethnomedizinisches Zentrum e.V. (EMZ). MiMi-Reha: Implementierung und Evaluation eines Info-Angebotes für MigrantInnen zur medizinischen Reha auf Basis der ‚MiMi-Kampagnentechnologie‘: Das Gesundheitsprojekt Mit Migranten für Migranten - Abschlussbericht -; 2017 [cited 2020 Mar 1]. Available from: http://forschung.deutsche-rentenversicherung.de/ForschPortalWeb/ressource?key=MiMi_REHA_Abschlussbericht.pdf. Accessed 1 March 2020.

[52] Nowik D, Bergman J, Markin K, Reißmann L, Salman R, Gutenbrunner C. Veränderungen subjektiver Zugangsbarrieren und Antragsintention zur Rehabilitation von MigrantInnen – Abschließende Ergebnisse aus MiMi-Reha. In: Deutsche Rentenversicherung Bund, editor. 26. Rehabilitationswissenschaftliches Kolloquium Deutscher Kongress für Rehabilitationsforschung: Prävention und Rehabilitation in Zeiten der Globalisierung vom 20. bis 22. März 2017 in Frankfurt am Main; Sonderausgabe. Berlin; 2017. p. 86–8 [DRV-Schriften; vol. 111] [cited 2020 Mar 6]. Available from: http://forschung.deutsche-rentenversicherung.de/ForschPortalWeb/ressource?key=tagungsband_26_reha_kolloqu.pdf. Accessed 6 March 2020.

[53] Reissmann, L.-M., Schwarz, B., Markin, K., Salman, R., Gutenbrunner, C. Ein Wegweiser für Migranten in die medizinische Rehabilitation der Deutschen Rentenversicherung. In: Deutsche Rentenversicherung Bund, editor. 24. Rehabilitationswissenschaftliches Kolloquium Deutscher Kongress für Rehabilitationsforschung: Psychische Störungen – Herausforderungen für Prävention und Rehabilitation vom 16. bis 18. März 2015 in Augsburg; 2015. p. 191–2 [DRV Schriften; vol. 107] [cited 2020 Mar 6]. Available from: http://forschung.deutsche-rentenversicherung.de/ForschPortalWeb/ressource?key=tagungsband_24_reha_kolloqu.pdf. Accessed 6 March 2020.

[54] Kohler M, Ziese T. Telefonischer Gesundheitssurvey des Robert-Koch-Instituts zu chronischen Krankheiten und ihren Bedingungen: Deskriptiver Ergebnisbericht. Berlin; 2004. Beiträge zur Gesundheitsberichterstattung des Bundes.

[55] Maier C. Migration und rehabilitative Versorgung in Deutschland: Ein Vergleich der Inanspruchnahme von Leistungen der medizinischen Rehabilitation und eines Indikators für Rehabilitationserfolg zwischen Rehabilitanden türkischer und nicht-türkischer Abstammung; 2008 [. (Veröffentlichungsreihe des Zentrums für Versorgungsforschung, Fakultät für Gesundheitswissenschaften, Universität Bielefeld) [cited 2020 Mar 3]. Available from: https://www.uni-bielefeld.de/gesundhw/zfv/maier.pdf. Accessed 3 Mar 2020.

[56] Ritter S, Dannenmaier J, Jankowiak S, Kaluscha R, Krischak G (2018). Implantation einer Hüft- oder Knietotalendoprothese und die Inanspruchnahme einer Anschlussrehabilitation. Rehabilitation.

[57] Schröder CC, Dyck M, Breckenkamp J, Hasselhorn HM, Du Prel J-B (2020). Utilisation of rehabilitation services for non-migrant and migrant groups of higher working age in Germany - results of the lidA cohort study. BMC Health Serv Res.

[58] Spallek L, Yilmaz-Aslan Y, Klein-Ellinghaus F, Gök Y, Zeeb H, Kolip P (2017). Deficits in psycho-oncological care among Turkish immigrant women with breast cancer in Germany – an interview study. IJPR.

[59] Yilmaz-Aslan Y, Spallek L, Gök Y, Kolip P, Spallek J. Krankheitsvorstellungen und Behandlungserwartungen nach der Diagnose Brustkrebs: Die besondere Situation türkischer Frauen. Dresden; 20.-22. November. (12. Jahrestagung der Arbeitsgemeinschaft für Psychoonkologie (PSO)).

[60] Zollmann P, Pimmer V, Rose AD, Erbstosser S (2016). Comparison of psychosomatic rehabilitation for German and foreign patients. Rehabilitation (Stuttg).

[61] Rommel A, Saß AC, Born S, Ellert U (2015). Die gesundheitliche Lage von Menschen mit Migrationshintergrund und die Bedeutung des sozioökonomischen Status: Erste Ergebnisse der Studie zur Gesundheit Erwachsener in Deutschland (DEGS1). Bundesgesundheitsbl Gesundheitsforsch Gesundheitsschutz.

[62] Schenk L, Neuhauser H, Ellert U, Poethko-Müller C, Kleiser C, Mensink G. Kinder-und Jugendgesundheitssurvey (KiGGS 2003-2006): Kinder und Jugendliche mit Migrationshintergrund in Deutschland 2008 [cited 2020 Mar 3]. Available from: https://www.rki.de/DE/Content/Gesundheitsmonitoring/Gesundheitsberichterstattung/GBEDownloadsB/KiGGS_migration.pdf?__blob=publicationFile. Accessed on 3 March 2020.

[63] Englum BR, Villegas C, Bolorunduro O, Haut ER, Cornwell EE, Efron DT (2011). Racial, ethnic, and insurance status disparities in use of posthospitalization care after trauma. J Am Coll Surg.

[64] Ellis C, Egede LE (2009). Racial/ethnic differences in poststroke rehabilitation utilization in the USA. Expert Rev Cardiovasc Ther.

[65] Devaux M, Lopper M de. Income-related inequalities in health service utilisation in 19 OECD countries, 2008–2009. Paris; 2012. OECD Health Working Paper 58. 10.1787/18152015.

[66] Department of Health UK Government. Tackling health inequalities. A programme for action; 2005 [cited 2020 Feb 23]. Available from: http://www.euro.who.int/__data/assets/pdf_file/0005/124529/E87934.pdf, Accessed 23 Feb 2020.

[67] Elrod CS, DeJong G (2008). Determinants of utilization of physical rehabilitation services for persons with chronic and disabling conditions: an exploratory study. Arch Phys Med Rehabil.

[68] Brzoska P, Razum O (2017). Herausforderungen einer diversitätssensiblen Versorgung in der medizinischen Rehabilitation. Rehabilitation (Stuttg).

[69] Luckert H. Statistikdaten der gesetzlichen Rentenversicherung–ein grober Überblick. In: Deutsche Rentenversicherung Bund, editor. Das Forschungsdatenzentrum der gesetzlichen Rentenversicherung (FDZ-RV) im Aufbau. Berlin; 2004 [DRV Schriften; vol. 24].

[70] Nielsen SS, Krasnik A, Rosano A (2009). Registry data for cross-country comparisons of migrants' healthcare utilization in the EU: a survey study of availability and content. BMC Health Serv Res.

[71] Dyck M, Wenner J, Wengler A, Bartig S, Fischer F, Wandschneider L (2019). Migration und Gesundheit in Deutschland – eine Bestandsaufnahme der Datenquellen. Bundesgesundheitsbl Gesundheitsforsch Gesundheitsschutz.

[72] Saß A-C, Grüne B, Brettschneider A-K, Rommel A, Razum O, Ellert U (2015). Beteiligung von Menschen mit Migrationshintergrund an Gesundheitssurveys des Robert Koch-Instituts. Bundesgesundheitsbl Gesundheitsforsch Gesundheitsschutz.

