# Supplementary material for: Utilisation of medical rehabilitation services by persons of working age with a migrant background, in comparison to non-migrants: a scoping review
Source: Public Health Rev. 2020 Aug 3;41:17. doi: 10.1186/s40985-020-00134-5 (PMC7397664; doi:10.1186/s40985-020-00134-5)
Supplement: Supplementary file 1 — Additional file 1: Table S1. Studies, technical information to answer the review question. Table S2. Studies, content-related information to answer the review question [file 40985_2020_134_MOESM1_ESM.pdf]

Additional file 1

S1 Studies, content-related information to answer the review question

| No. | Authors | Linked source | Intervention | | | Differentiation of the Migration status | | | | | Groups (origin) with and without migration background | | | | | | | Medical indications | Main results | | | | | | | |
|---|---|---|---|---|---|---|---|---|---|---|---|---|---|---|---|---|---|---|---|---|---|---|---|---|---|---|
| | | | | | | | | | | | | | | | | | | | Research focus | | | | | | | Differences between groups |
| | | | | | | | | | | | | non-German | | | | | | | | | | | | | | |
| | | | During rehabilitation /complete | Before rehabilitation | After rehabilitation (incl. discharge) | Nationality | Place of birth | Migration status/ migration experience/ Immigrated after 1949 | Native/ spoken language | Other (e.g. PMB and non-PMB, not specified) | German | Turkish | Former Yugoslavian | Citizens of the former Soviet-Union | Resettler | Mediterranean (S, G, P, I) | Others (e.g. non-Turkish, non-German, EU-nationals) | | Utilisation of rehabilitation services | Barriers to rehabilitation services | Satisfaction with the utilisation of rehabilitation ser- | Perceptions/ expectations and needs/ intentions | Rehabilitation (treatment) success/ treatment outcome | Work-related performance | Return to work | |
| 1. | Aksakal et al. 2018 [1] | [1] | x | | | | | | | x | | | | | | | o | | | | x | x | | | | Insufficient knowledge, treatment desires, language barriers for PMB compared to non-PMB |
| 2. | Brause et al. 2010 [2] | [3, 4] | x | | | x* | | | | x | | x | | | | | x | 1-6 | x | x | | x | x | x | | Turkish PMB: lower rehabilitation success and work ability for musculoskeletal/connective tissue, mental illnesses metabolism/digestion and other (respiratory diseases) than for non-PMB<br>Barriers for Turkish PMB (access, knowledge, language, culture) |

[1] Personal Communication with Aksakal and Yilmaz-Aslan (March 2020)

| No. | Authors | Linked source | Intervention | | | Differentiation of the Migration status | | | | | | Groups (origin) with and without migration background | | | | | | | | Medical indications | Main results | | | | | | | |
|---|---|---|---|---|---|---|---|---|---|---|---|---|---|---|---|---|---|---|---|---|---|---|---|---|---|---|---|
| | | | | | | | | | | | | | non-German | | | | | | | | Research focus | | | | | | | Differences between groups |
| | | | During rehabilitation /complete | Before rehabilitation | After rehabilitation (incl. discharge) | Nationality | Place of birth | Migration status/ migration experience/ Immigrated after 1949 | Native/ spoken language | Other (e.g. PMB and non-PMB, not specified) | German | Turkish | Former Yugoslavian | Citizens of the former Soviet-Union | Resettler | Mediterranean (S, G, P, I) | Others (e.g. non-Turkish, non-German, EU-nationals) | | Utilisation of rehabilitation services | Barriers to rehabilitation services | Satisfaction with the utilisation of rehabilitation ser- | Perceptions/ expectations and needs/ intentions | Rehabilitation (treatment) success/ treatment outcome | Work-related performance | Return to work | |
| 3. | Brzoska et al. 2019 [5] | [6] | | | x | x | x | | x | x | x | | | | | | x | | x | | | | | | | German nationals (non-PMB and PMB) and non-German nationals did not differ in their utilisation of rehabilitation |
| 4. | Brzoska et al. 2019 [7] | | | | | x | | | | X[3] | x | | | | x | | x | | x | | | | | | | Foreigners (PMB) compared to Germans without Resettler status had a lower chance to use medical rehabilitation<br>Resettler had a higher chance of using rehabilitation compared to foreigners |
| 5. | Brzoska et al. 2017 [8] | | x | | | x | | | | | x | x | x | | | x | x | 1-3,6 | | | x | | | | | Lower probability for satisfaction with rehabilitation for Turkish nationals (PMB)<br>Other foreign nationals were as satisfied as German nationals (non-PMB) |
| 6. | Brzoska et al. 2016 [9] | | x | | | x | | | | | x | x | x | | | x | x | | | | | | x | x | | Non-Germans report less favourable outcomes, Turkish and former Yugoslavian origin have a higher chance for a poor treatment outcome than patients from Mediterranean countries |

| No. | Authors | Linked source | Intervention: During rehabilitation /complete | Intervention: Before rehabilitation | Intervention: After rehabilitation (incl. discharge) | Differentiation of the Migration status: Nationality | Differentiation of the Migration status: Place of birth | Differentiation of the Migration status: Migration status/ migration experience/ Immigrated after 1949 | Differentiation of the Migration status: Native/ spoken language | Differentiation of the Migration status: Other (e.g. PMB and non-PMB, not specified) | Groups (origin) with and without migration background: German | Groups (origin) with and without migration background: non-German: Turkish | Groups (origin) with and without migration background: non-German: Former Yugoslavian | Groups (origin) with and without migration background: non-German: Citizens of the former Soviet-Union | Groups (origin) with and without migration background: non-German: Resettler | Groups (origin) with and without migration background: non-German: Mediterranean (S, G, P, I) | Groups (origin) with and without migration background: Others (e.g. non-Turkish, non-German, EU-nationals) | Medical indications | Main results: Research focus: Utilisation of rehabilitation services | Main results: Research focus: Barriers to rehabilitation services | Main results: Research focus: Satisfaction with the utilisation of rehabilitation ser- | Main results: Research focus: Perceptions/ expectations and needs/ intentions | Main results: Research focus: Rehabilitation (treatment) success/ treatment outcome | Main results: Research focus: Work-related performance | Main results: Research focus: Return to work | Main results: Differences between groups |
|---|---|---|---|---|---|---|---|---|---|---|---|---|---|---|---|---|---|---|---|---|---|---|---|---|---|---|
| 7. | Brzoska et al. 2012 [10] | [11] | x | x | x | | x | | x | | x | x | x | | | x | x | | | | | | x | x | | Non-Germans showed a higher chance for low occupational performance after completing the rehabilitation a lower effectiveness of rehabilitation for Turkish and former Yugoslavian compared to Germans |
| 8. | Brzoska et al. 2010 [11] | [12–18] | x | x | x | x | x | | x | x | x | x | x | | x | x | x | 1-6 | x | x | | x | x | X | | Non-Germans less utilised rehabilitation than Germans,<br>Foreigner have a higher chance than Germans for occupational diseases, lower rehabilitation occupational performance and effectiveness after rehabilitation, PMB had barriers for utilisation compared to non-PMB: expectations, information deficit, missing intention to apply, language and culture |

| No. | Authors | Linked source | Intervention | | | Differentiation of the Migration status | | | | | Groups (origin) with and without migration background | | | | | | | Medical indications | Main results | | | | | | | |
|---|---|---|---|---|---|---|---|---|---|---|---|---|---|---|---|---|---|---|---|---|---|---|---|---|---|---|
| | | | | | | | | | | | | non-German | | | | | | | Research focus | | | | | | | Differences between groups |
| | | | During rehabilitation /complete | Before rehabilitation | After rehabilitation (incl. discharge) | Nationality | Place of birth | Migration status/ migration experience/ Immigrated after 1949 | Native/ spoken language | Other (e.g. PMB and non-PMB, not specified) | German | Turkish | Former Yugoslavian | Citizens of the former Soviet-Union | Resettler | Mediterranean (S, G, P, I) | Others (e.g. non-Turkish, non-German, EU-nationals) | | Utilisation of rehabilitation services | Barriers to rehabilitation services | Satisfaction with the utilisation of rehabilitation ser- | Perceptions/ expectations and needs/ intentions | Rehabilitation (treatment) success/ treatment outcome | Work-related performance | Return to work | |
| 9. | Erbstößer/ Zollmann 2015 [19] | | x | x | x | x | | | | x | x | x | x | x | | x | x | 1-6 | x | | | | x | x | x | Non-Germans less utilised rehabilitation (heterogeneous for nationalities), had less often a full work performance after rehabilitation than Germans,<br>Differences in indications e.g. for musculoskeletal rehabilitation: patients from former Soviet-Union utilised more than other nationals and Germans |
| 10. | Göbber et al. 2010 [20] | [21] | | | x | x | | | x | x | | x | | | | | x | 2-5 | | | | x | x | x | x | PMB were pension-oriented, desired for gender-specific treatment<br>PMB had a shorter treatment-duration, negative work performance (subjective), more mental and somatoform illnesses than non-PMB |
| 11. | Gruner et al. 2012 [22] | | x | x | x | | | x | | x | x | x | x | x | | x | | 5 | | | | x | x | x | | PMB had a higher frequency of diseases, less work performance before and after rehabilitation (subjective), pension desire, are sicker at the beginning of rehabilitation, men (non-PMB) benefitted less |

| No. | Authors | Linked source | Intervention | | | Differentiation of the Migration status | | | | | Groups (origin) with and without migration background | | | | | | | Medical indications | Main results: Research focus | | | | | | | Main results: Differences between groups |
|---|---|---|---|---|---|---|---|---|---|---|---|---|---|---|---|---|---|---|---|---|---|---|---|---|---|---|
| | | | During rehabilitation /complete | Before rehabilitation | After rehabilitation (incl. discharge) | Nationality | Place of birth | Migration status/ migration experience/ Immigrated after 1949 | Native/ spoken language | Other (e.g. PMB and non-PMB, not specified) | German | non-German: Turkish | non-German: Former Yugoslavian | non-German: Citizens of the former Soviet-Union | non-German: Resettler | non-German: Mediterranean (S, G, P, I) | Others (e.g. non-Turkish, non-German, EU-nationals) | | Utilisation of rehabilitation services | Barriers to rehabilitation services | Satisfaction with the utilisation of rehabilitation ser- | Perceptions/ expectations and needs/ intentions | Rehabilitation (treatment) success/ treatment outcome | Work-related performance | Return to work | |
| 12. | Höhne 2007a [23] | | x | | | X | | | | x | x | x | | | | | | 1-3,5-6 | x | | | | | x | | Disabled pensioners (PMB) utilised less rehabilitation services in the last five years before retirement |
| 13. | Höhne / Schubert 2007b [24] | | x | | x | x | | | | | x | x | | | | | X | 1-6 | x | | | | | x | | Differences in the medical rehabilitation benefits between Germans and Non-Germans in the last 5 years before retirement: Non-German had a higher incapacity for work, mental illnesses affect non-Germans more than Germans |
| 14. | Höhne et al. 2007c [25] | | x | | x | x | | | | | x | | | | | | X | 1-3,5-6 | x | | | | x | x | | Differences in the utilisation of rehabilitation: Germans utilised more services of rehabilitation than PMB |
| 15. | Jankowiak et al. 2018 [26] | | x | | | x | | | | x | x | | | | | | x | | x | | | | | | | Medical rehabilitation was less utilised by non-Germans than Germans, Applications for rehabilitation were lower for non-Germans than Germans |

| No. | Authors | Linked source | Intervention | | | Differentiation of the Migration status | | | | | Groups (origin) with and without migration background | | | | | | | Medical indications | Main results: Research focus | | | | | | | Main results: Differences between groups |
|---|---|---|---|---|---|---|---|---|---|---|---|---|---|---|---|---|---|---|---|---|---|---|---|---|---|---|
| | | | During rehabilitation /complete | Before rehabilitation | After rehabilitation (incl. discharge) | Nationality | Place of birth | Migration status/ migration experience/ Immigrated after 1949 | Native/ spoken language | Other (e.g. PMB and non-PMB, not specified) | German | non-German: Turkish | non-German: Former Yugoslavian | non-German: Citizens of the former Soviet-Union | non-German: Resettler | non-German: Mediterranean (S, G, P, I) | Others (e.g. non-Turkish, non-German, EU-nationals) | | Utilisation of rehabilitation services | Barriers to rehabilitation services | Satisfaction with the utilisation of rehabilitation ser- | Perceptions/ expectations and needs/ intentions | Rehabilitation (treatment) success/ treatment outcome | Work-related performance | Return to work | |
| 16. | Kaluscha et al. 2011 [27] | | x | | | x | | | | | x | x | x | | | x | x | 1-5 | x | | | | | | | Turkish nationals utilise rehabilitation more often due to mental illnesses than Germans |
| 17. | Kessemaier et al. 2019 [28] | | | | | x | x | | x | x | | | | | | | x | | | | x | | | | | PMB showed severe symptoms and were not as satisfied with the rehabilitation as non-PMB |
| 18. | MHH/ EMZ e.V. 2017 [29] | [30–32] | | x | x | | | x | | x | x | x | | x | | | x | | | x | | x | | | | Barriers to utilise rehabilitation: migrant specific (information deficit), person-related barriers (language), systematic-related barriers (bureaucracy), barriers independent of migrant status (fear of job loss),<br>Increased application intention through the campaign for PMB |

| No. | Authors | Linked source | Intervention | | | Differentiation of the Migration status | | | | | Groups (origin) with and without migration background | | | | | | | Medical indications | Main results | | | | | | | |
|---|---|---|---|---|---|---|---|---|---|---|---|---|---|---|---|---|---|---|---|---|---|---|---|---|---|---|
| | | | | | | | | | | | | | | | | | | | Research focus | | | | | | | Differences between groups |
| | | | | | | | | | | | | non-German | | | | | | | | | | | | | | |
| | | | During rehabilitation /complete | Before rehabilitation | After rehabilitation (incl. discharge) | Nationality | Place of birth | Migration status/ migration experience/ Immigrated after 1949 | Native/ spoken language | Other (e.g. PMB and non-PMB, not specified) | German | Turkish | Former Yugoslavian | Citizens of the former Soviet-Union | Resettler | Mediterranean (S, G, P, I) | Others (e.g. non-Turkish, non-German, EU-nationals) | | Utilisation of rehabilitation services | Barriers to rehabilitation services | Satisfaction with the utilisation of rehabilitation ser- | Perceptions/ expectations and needs/ intentions | Rehabilitation (treatment) success/ treatment outcome | Work-related performance | Return to work | |
| 19. | Kohler/ Ziese 2004 [33] | | x | x | x | x | x | x | | | x | | | | | | x | | x | | | | | | | PMB utilised rehabilitation less often than Germans |
| 20. | Maier 2008 [34] | | x | | x | x* | | | | x | | x | | | | | x | 1-3,5 | x | | | | x | | | Turkish rehabilitants utilised more rehabilitation services than the non-Turkish, health didn't improve as much as in non-Turkish, differences in indications: more musculoskeletal and mental illnesses for Turkish PMB |
| 21. | Pfeiffer et al. 2010 [21] | [20] | | | | x | | x | | x | x | x | x | x | x | x | x | 2-4 | | | | x | x | | x | PMB were pension-oriented, had a shorter treatment-duration, more mental and somatoform illnesses than non-PMB, other treatment expectations than non-PMB |
| 22. | Ritter et al. 2017 [35] | | x | x | x | x | | | | | x | | | | | | X | 2 | x | | | | | | | Lower chance of utilisation for foreign nationals with hip and knee arthroplasty compared to Germans |

| No. | Authors | Linked source | Intervention | | | Differentiation of the Migration status | | | | | Groups (origin) with and without migration background | | | | | | | Medical indications | Main results | | | | | | | |
|---|---|---|---|---|---|---|---|---|---|---|---|---|---|---|---|---|---|---|---|---|---|---|---|---|---|---|
| | | | | | | | | | | | | non-German | | | | | | | Research focus | | | | | | | Differences between groups |
| | | | During rehabilitation /complete | Before rehabilitation | After rehabilitation (incl. discharge) | Nationality | Place of birth | Migration status/ migration experience/ Immigrated after 1949 | Native/ spoken language | Other (e.g. PMB and non-PMB, not specified) | German | Turkish | Former Yugoslavian | Citizens of the former Soviet-Union | Resettler | Mediterranean (S, G, P, I) | Others (e.g. non-Turkish, non-German, EU-nationals) | | Utilisation of rehabilitation services | Barriers to rehabilitation services | Satisfaction with the utilisation of rehabilitation ser- | Perceptions/ expectations and needs/ intentions | Rehabilitation (treatment) success/ treatment outcome | Work-related performance | Return to work | |
| 23. | Schröder et al. 2020 [36] | | x | | | x | x | x | | | x | | | | | | x | | x | | | | | | | First-generation migrants had a lower chance of utilising outpatient rehabilitation than non-migrants<br>No differences between first- and second-generation migrants and non-migrants for any rehabilitation |
| 24. | Yilmaz-Aslan et al. 2017 [37] | [38] | x | x | | | | | x | x | x | x | | | | | | 1 | x | x | x | x | | | | Turkish PMB in comparison to Germans: higher need for support (information and emotional), psycho-oncological care was rarely utilised, barriers for Turkish-PMB e.g. information deficit, culture, language and prejudice |
| 25. | Zollmann et al. 2016 [39] | | x | x | x | x | | | | | x | x | x | | | | x | 5 | x | | | | x | x | x | Turkish PMB is largest group in psychosomatic rehabilitation (year 2012), were sicker at the beginning of rehabilitation than non-Turkish nationals, reintegration into working life is less successful for them |

Key to Table S1

| Symbols and Abbreviations | Explanation |
|---|---|
| * | Differentiation of the migration status included using a name-based algorithm |
| x | Information available |
| o | Information not available |
| PMB | Persons with a migrant background |
| non-PMB | Persons without a migrant background |
| Medical indications numbers:<br>1<br>2<br>3<br>4<br>5<br>6 | <br>neoplasms<br>muscles/connective tissue<br>cardiovascular<br>metabolism/digestion<br>mental illnesses (incl. addiction)<br>other (not described, e.g. respiratory diseases) |

Table S2 Studies, technical information to answer the review question

| No. | Authors | Linked source | Publication Type | Type of care | | | Setting | Year of the data | Study design | | | | | | Methods | | | | | | | Sample size | Service Provider | |
|---|---|---|---|---|---|---|---|---|---|---|---|---|---|---|---|---|---|---|---|---|---|---|---|---|
| | | | | Inpatient | Outpatient | Other/both | | | Cross-sectional | Longitudinal | Survey | Evaluation (e.g. quasi-experimental) | Cohort | Qualitative | Primary data | Secondary data | data analysis methods | Focus groups | Structured interviews | Semi-structured (problem-centred) inter-views | Expert interviews | | German statutory pension insurance | Other |
| 1. | Aksakal et al. 2018 [1] | [2] | Conference ab-stract *Rehabilita-tion science collo-quium of the Ger-man statutory pension insurance* | x | | | North Rhine-Westphalia Germany | 2016 | | X | | $x_B$ | | $x_A$ | x | | i,d,c | | $x_B$ | | $x_A$ | **A** n= 12 rehabilitants, 16 employees<br>**B** Intervention T0 n= 89 rehabilitants<br>Control T0 n=164 rehabili-tants<br>Intervention T1 n= 150 rehabilitants<br>Control T1 n=150 rehabili-tants<br><br>n= 15 rehabilitants ,10 employees | | o |
| 2. | Brause et al. 2010 [2] | [3, 4] | Report | | | x | Rhineland & North Rhine-Westphalia Germany | **A** 2000-2006<br>**B** 2008-2009 | x | | | | | $x_B$ | | $x_A$ | d,i,c | $x_B$ | | x | $x_B$ | **A** n= 363 855 (4.8% Turk-ish)<br>**B** n= 19 rehabilitants, 12 employees | x | |

[2] Personal Communication

| No. | Authors | Linked source | Publication Type | Type of care | | | Setting | Year of the data | Study design | | | | | | Methods | | | | | | | Sample size | Service Provider | |
|---|---|---|---|---|---|---|---|---|---|---|---|---|---|---|---|---|---|---|---|---|---|---|---|---|
| | | | | Inpatient | Outpatient | Other/both | | | Cross-sectional | Longitudinal | Survey | Evaluation (e.g. quasi-experimental) | Cohort | Qualitative | Primary data | Secondary data | data analysis methods | Focus groups | Structured interviews | Semi-structured (problem-centred) inter-views | Expert interviews | | German statutory pension insurance | Other |
| 3. | Brzoska et al. 2019 [5] | [6] | Original research paper *Zeitschrift für Gerontologie und Geriatrie* | | | x | Germany | 2015 | x | x | x | | x | | | x | i,d | | | | | n= retrospective 17 369 persons retired due to disability | x | |
| 4. | Brzoska et al. 2019 [7] | | Original research paper *Die Rehabilitation* | | | x | Germany | Sociomedical panel III 2013 extended with data of the GPI | | x | x | | x | | x | x | i,d | | | | | n= 2 413<br>2,7% foreign nationals<br>4,4% German nationals with migrant background | | o |
| 5. | Brzoska et al. 2017 [8] | | Original research paper *BMJ Open* | x | | | Hospitals of the GPI | 2007-2011 | x | | x | | | | | x | i,d | | | | | n= 274 513 & 624 hospitals | | |
| 6. | Brzoska et al. 2016 [9] | | Original research paper *BMC Health Services Research* | x | | | Hospitals of the GPI | 2007-2011 | x | | x | | | | | x | i,d | | | | | n= 274 513 and 624 hospitals<br>(PMB n=11 329) | x | |

| No. | Authors | Linked source | Publication Type | Type of care | | | Setting | Year of the data | Study design | | | | | | Methods | | | | | | | Sample size | Service Provider | |
|---|---|---|---|---|---|---|---|---|---|---|---|---|---|---|---|---|---|---|---|---|---|---|---|---|
| | | | | Inpatient | Outpatient | Other/both | | | Cross-sectional | Longitudinal | Survey | Evaluation (e.g. quasi-experimental) | Cohort | Qualitative | Primary data | Secondary data | data analysis methods | Focus groups | Structured interviews | Semi-structured (problem-centred) inter-views | Expert interviews | | German statutory pension insurance | Other |
| 7. | Brzoska et al. 2012 [10] | [11] | Original research paper *Gesund-heitswesen* | | | x | GPI | 2006 | x | | | | | | | x | i,d | | | | | n= 634 529<br>(PMB n= 31 177) | x | |

| No. | Authors | Linked source | Publication Type | Type of care | | | Setting | Year of the data | Study design | | | | | | Methods | | | | | | | Sample size | Service Provider | |
|---|---|---|---|---|---|---|---|---|---|---|---|---|---|---|---|---|---|---|---|---|---|---|---|---|
| | | | | Inpatient | Outpatient | Other/both | | | Cross-sectional | Longitudinal | Survey | Evaluation (e.g. quasi-experimental) | Cohort | Qualitative | Primary data | Secondary data | data analysis methods | Focus groups | Structured interviews | Semi-structured (problem-centred) inter-views | Expert interviews | | German statutory pension insurance | Other |
| 8. | Brzoska et al. 2010 [11] | [12–18] | Report | | | x | **I.**<br>**A + B** German Socio-Economic Panel data<br>**II.** statutory accident insurance data + data from the German working agency<br>**III.** GPI<br>**IV.** qualitative data | **I. A** 2002-2004<br>**B** 2005-2007<br>**II.** 1999-2007<br>**III.** 2006<br>**IV.** o | x | x | x | | | $x_{IV}$ | | $x_{I-III}$ | d,i,c | $x_{IV}$ | $x_{IV}$ | $x_{IV}$ | $x_{IV}$ | **I. A** n= 19 521 persons from German households (3 008 PMB)<br>**B** n= 17 176 persons from German households (2 551 PMB)<br>**II.** n= o rehabilitation services in German statutory accident insurance<br>**III.** n= 634 529 rehabilitants<br>4.9% PMB<br>**IV.** n=15 experts, PMB: n=22 (18 Turkish origin, 4 Resettler), rehabilitants (medical), n=14 (9 Turkish origin, 5 Resettler) rehabilitants (occupational) | x | x |

| No. | Authors | Linked source | Publication Type | Type of care | | | Setting | Year of the data | Study design | | | | | | Methods | | | | | | | Sample size | Service Provider | |
|---|---|---|---|---|---|---|---|---|---|---|---|---|---|---|---|---|---|---|---|---|---|---|---|---|
| | | | | Inpatient | Outpatient | Other/both | | | Cross-sectional | Longitudinal | Survey | Evaluation (e.g. quasi-experimental) | Cohort | Qualitative | Primary data | Secondary data | data analysis methods | Focus groups | Structured interviews | Semi-structured (problem-centred) inter-views | Expert interviews | | German statutory pension insurance | Other |
| 9. | Erbstößer/ Zoll-mann 2015 [19] | | Original research paper *RVaktuell* | | | x | GPI | 2012 | x | | | | | | | x | d | | | | | n= 729 244 | x | |
| 10. | Göbber et al. 2010 [20] | [21] | Original research paper *Zeitschrift für Psychiatrie, Psychologie und Psychotherapie* | x | | | Clinic "Ha-senbach" | 2006-2009 | x | | x | | | | x | | d,i | | | | | n= 170 Turkish<br>n= 351 non- PMB | x | |
| 11. | Gruner et al. 2012 [22] | | Original research paper *Zeitschrift für Psychosomati-sche Medizin und Psychotherapie* | x | | | Clinic "Schloss Bad Buchau" | 2008-2010 | | x | | | x | | x | | d,i | | | | | n= 75 PM Cases (30% ex-Soviet Union, 28% former Yugoslavia, 13% Italy, 7% Turkey),<br>non- PMB -Control group n=123 | x | |
| 12. | Höhne 2007a [23] | | Other publication type (Congress documentation) *bundesweiter Ar-mut und Gesund-heitskongress 2006* | | | x | GPI | **A** 2003 and **B** 2004 | x | | | | | | | x | d | | | | | **A** n= 89 911<br>and **B** n= 88 852<br>only new pensioners and pensioners with reduction in earning capacity | x | |

| No. | Authors | Linked source | Publication Type | Type of care | | | Setting | Year of the data | Study design | | | | | | Methods | | | | | | | Sample size | Service Provider | |
|---|---|---|---|---|---|---|---|---|---|---|---|---|---|---|---|---|---|---|---|---|---|---|---|---|
| | | | | Inpatient | Outpatient | Other/both | | | Cross-sectional | Longitudinal | Survey | Evaluation (e.g. quasi-experimental) | Cohort | Qualitative | Primary data | Secondary data | data analysis methods | Focus groups | Structured interviews | Semi-structured (problem-centred) inter-views | Expert interviews | | German statutory pension insurance | Other |
| 13. | Höhne / Schubert 2007b [24] | | Other publication type (Essay from *DRV-Schriften) (Writings of the German statutory pension insurance)* | | | x | GPI from Braun-schweig and Hannover | 2003 | x | | | | | | | x | d,i | | | | | n= 100 298, (n= 89 911 foreigners and Turkish nationals) | x | |
| 14. | Höhne et al. 2007c [25] | | Conference ab-stract *Rehabilitation science colloquium of the German statutory pension insurance* | | | x | GPI from Braun-schweig and Hannover | 2003 | x | | | | | | | x | d,i | | | | | n= 100 298 from that a 10% sample | x | |
| 15. | Jankowiak et al. 2018 [26] | | Conference ab-stract *Rehabilitation science colloquium of the German statutory pension insurance* | | | x | GPI from Baden-Württem-berg | 2008-2015 | | x | | | | | | x | d,i | | | | | Population from birth year 1963 | x | |

| No. | Authors | Linked source | Publication Type | Type of care | | | Setting | Year of the data | Study design | | | | | | Methods | | | | | | | Sample size | Service Provider | |
|---|---|---|---|---|---|---|---|---|---|---|---|---|---|---|---|---|---|---|---|---|---|---|---|---|
| | | | | Inpatient | Outpatient | Other/both | | | Cross-sectional | Longitudinal | Survey | Evaluation (e.g. quasi-experimental) | Cohort | Qualitative | Primary data | Secondary data | data analysis methods | Focus groups | Structured interviews | Semi-structured (problem-centred) inter-views | Expert interviews | | German statutory pension insurance | Other |
| 16. | Kaluscha et al. 2011 [27] | | Conference ab-stract *Rehabilitation science colloquium of the German statutory pension insurance* | | | x | GPI | 2006-2008 | x | | | | | | | x | i | | | | | n= 192 459 from that<br>n= 42,317 with mental illnesses<br>18% Mediterranean,<br>20.4% former Yugoslavian,<br>31.4% Turkish,<br>Others 21.4% | | |
| 17. | Kessemeier et al. 2019 [28] | | Original research paper in *Phys Med Rehab Kuror* | x | | | two rehabili-tation hospi-tals "Reha-zentrum Oberharz" and "Reha-bilita-tionsklinik Bad Pyr-mont" | 2017 | | x | | | x | | | x | i | | | | | n=3 169, matched sample n=1 468<br><br>23.2% PMB (5.4% non-German nationality,<br>11.2% non-German moth-er tongue, 22,1 % other place of birth )<br>76,8% non-PMB | o | |

| No. | Authors | Linked source | Publication Type | Type of care | | | Setting | Year of the data | Study design | | | | | | Methods | | | | | | | Sample size | Service Provider | |
|---|---|---|---|---|---|---|---|---|---|---|---|---|---|---|---|---|---|---|---|---|---|---|---|---|
| | | | | Inpatient | Outpatient | Other/both | | | Cross-sectional | Longitudinal | Survey | Evaluation (e.g. quasi-experimental) | Cohort | Qualitative | Primary data | Secondary data | data analysis methods | Focus groups | Structured interviews | Semi-structured (problem-centred) inter-views | Expert interviews | | German statutory pension insurance | Other |
| 18. | MHH/ EMZ e.V. 2017 [29] | [30–32] | Report | | | x | Lübeck, Hannover, Bremen Germany | 2012-2014 | | x | | x | | x | X | | d,i, c | X | X | | x | **A** n= 10 Russian and Turkish<br>**B** n=1 371 PMB<br>**C** n= 42<br>(n=7 rehab-experienced, n= 7 rehab- unexperienced, n= 10 German, n= 8 Russian speaking, n= 10 Turkish speaking) | (x) | |
| 19. | Kohler/ Ziese 2004 [33] | | Report | | | x | German housholds with landline | 2000-2003 | x | | x | | | | x | | d | | | | | n= 8 313 | x | x |
| 20. | Maier 2008 [34] | | Original research paper *Zentrum für Versorgungsforschung, Fakultät für Gesundheitswissenschaften, Universität Bielefeld* | | | x | GPI from Rhineland | 2000-2004 | | x | | | | | | x | d | | | | | n= 146 801 insured Turkish rehabilitants | x | |

| No. | Authors | Linked source | Publication Type | Type of care | | | Setting | Year of the data | Study design | | | | | | Methods | | | | | | | Sample size | Service Provider | |
|---|---|---|---|---|---|---|---|---|---|---|---|---|---|---|---|---|---|---|---|---|---|---|---|---|
| | | | | Inpatient | Outpatient | Other/both | | | Cross-sectional | Longitudinal | Survey | Evaluation (e.g. quasi-experimental) | Cohort | Qualitative | Primary data | Secondary data | data analysis methods | Focus groups | Structured interviews | Semi-structured (problem-centred) inter-views | Expert interviews | | German statutory pension insurance | Other |
| 21. | Pfeiffer et al. 2010 [21] | [20] | book chapter in *Neue Ansätze in der psychosomatischen Rehabilitation* | x | | | Clinic "Hasenbach" | 2006-2009 | x | | x | | | | x | | d | | | | | n= 274 PMB (153 Turkish origin, 17 Curdish origin, 39 Resettlers, 59 east European and 6 from other origin)<br>n= 351 non- PMB | x | |
| 22. | Ritter et al. 2017 [35] | | Original research paper *Die Rehabilitation* | x | x | x | | 2005-2010 | x | | x | | | | | x | d,i | | | | | n= 16 888 patients | x | x |
| 23. | Schröder et al. 2020 [36] | | Original Research paper *BMC Health Services Research* | | | x | Germany | 2011 | | x | | | x | | x | | d,i | | | | | n= 6 303 socially insured employees | x | |
| 24. | Yilmaz-Aslan et al. 2017 [37] | [38] | Original research paper *International Journal of Psychotherapy Practice and Research* | x | | | Bielefeld, Bremen and hospitals in *Bad Salzuflen* | **A-C** 2013-2014 | x | | | | | x | | | c | x A | | x B,C | | **A** Bielefeld, 2 German and 2 Turkish focus groups<br>**B** Bremen, n= 8 German and n= 8 Turkish women<br>**C** Bad Salzuflen, n= 8 German and n=8 Turkish women | | x |

| No. | Authors | Linked source | Publication Type | Type of care | | | Setting | Year of the data | Study design | | | | | | Methods | | | | | | | Sample size | Service Provider | |
|---|---|---|---|---|---|---|---|---|---|---|---|---|---|---|---|---|---|---|---|---|---|---|---|---|
| | | | | Inpatient | Outpatient | Other/both | | | Cross-sectional | Longitudinal | Survey | Evaluation (e.g. quasi-experimental) | Cohort | Qualitative | Primary data | Secondary data | data analysis methods | Focus groups | Structured interviews | Semi-structured (problem-centred) inter-views | Expert interviews | | German statutory pension insurance | Other |
| 25. | Zollmann et al. 2016 [39] | | Original research paper *Rehabilita-tion* | | | x | GPI | 2012 | x | | | | | | | x | d | | | | | n= 128 165<br>5.8 % PMB<br>2% Turkish | x | |

Key to Table S2

| Symbol | Explanation |
|---|---|
| I, II, III… | sub studies |
| A, B, C… | source of data is the same, different sample size or settings were used |
| x | Information available |
| o | Information not available |
| GPI | German statutory pension insurance data |
| PMB | Persons with a migrant background |
| non-PMB | Persons without a migrant background |
| quantitative data analysis methods | d descriptive analysis<br>i inductive analysis<br>c content analysis |

## References


1. Aksakal, T., Yilmaz-Aslan, Y., Akbulut, N., Razum, O., Brzoska, P. Umsetzung einer diversitätssensiblen Versorgung in Rehabilitationseinrichtungen. Ergebnisse einer exemplarischen Ist-Analyse. In: Deutsche Rentenversicherung Bund, editor. 27. Rehabilitationswissenschaftliches Kolloquium Deutscher Kongress für Rehabilitationsforschung: Rehabilitation bewegt! vom 26. bis 28. Februar 2018 in München; Sonderausgabe der DRV. Berlin; 2018. p. 512–4 [DRV Schriften; vol. 113]. [cited 2020 Mar 6]. Available from: http://forschung.deutsche-rentenversicherung.de/ForschPortalWeb/ressource?key=tagungsband_27_reha_kolloqu.pdf. Accessed 6 March 2020
2. Brause M, Reutin B, Schott T, Yilmaz-Aslan Y. Migration und gesundheitliche Ungleichheit in der Rehabilitation: Versorgungsbedarf und subjektive Bedürfnisse türkischer und türkisch-stämmiger Migrant(inn)en im System der medizinischen Rehabilitation: -Abschlussbericht -; 2010 [cited 2020 Mar 3]. Available from https://www.uni-bielefeld.de/gesundhw/zfv/endbericht.pdf. Access 3 March 2020
3. Brause M, Reutin B, Razum O, Schott T. Rehabilitation results of Turkish immigrants - an analysis of routine data from the Rhineland and Westfalia Pension Insurance. Rehabilitation (Stuttg). 2012;51:282–8. doi:10.1055/s-0031-1295448.
4. Brause M, Schott T. Reha-Inanspruchnahme und Erfolg bei Menschen mit türkischem Migrationshintergrund. Migration und medizinische Rehabilitation. Weinheim, Basel: Beltz Juventa Verlag. 2013:62–91.
5. Brzoska P, Spanier K, Bethge M. Potenziale des Dritten Sozialmedizinischen Panels für Erwerbspersonen (SPE-III) für die Forschung im Bereich Migration und Rehabilitation: Das Beispiel der Inanspruchnahme rehabilitativer Versorgung. Rehabilitation (Stuttg). 2019;58:385–91. doi:10.1055/a-0847-3234.
6. Brzoska P. Inanspruchnahme rehabilitativer Versorgung bei Menschen mit Migrationshintergrund. Untersuchungspotenziale des 'Dritten Sozialmedizinischen Panels für Erwerbspersonen' (SPE-III). In: Deutsche Rentenversicherung Bund, editor. 27. Rehabilitationswissenschaftliches Kolloqiuim Deutscher Kongress für Rehabilitationsforschung: Rehabilitation bewegt! vom 26. bis 28. Februar 2018 in München; Sonderausgabe. Berlin; 2018. p. 231–3 [DRV Schriften 113] [cited 2020 Mar 6]. Available from: http://forschung.deutsche-rentenversicherung.de/ForschPortalWeb/ressource?key=tagungsband_27_reha_kolloqu.pdf. Accessed 6 March 2020
7. Brzoska P, Razum O. Inanspruchnahme medizinischer Rehabilitation im Vorfeld der Erwerbsminderungsrente: Vergleich ausländischer und deutscher Staatsangehöriger unter besonderer Berücksichtigung von (Spät-)Aussiedler/-innen. Zeitschrift fur Gerontologie und Geriatrie. 2019;52:70–7. doi:10.1007/s00391-018-1448-y.
8. Brzoska P, Sauzet O, Yilmaz-Aslan Y, Widera T, Razum O. Satisfaction with rehabilitative health care services among German and non-German nationals residing in Germany: A cross-sectional study. BMJ Open. 2017;7:e015520. doi:10.1136/bmjopen-2016-015520.
9. Brzoska P, Sauzet O, Yilmaz-Aslan Y, Widera T, Razum O. Self-rated treatment outcomes in medical rehabilitation among German and non-German nationals residing in Germany: An exploratory cross-sectional study. BMC Health Serv Res. 2016;16:105. doi:10.1186/s12913-016-1348-z.
10. Brzoska P, Voigtlander S, Spallek J, Razum O. Die Nutzung von Routinedaten in der rehabilitationswissenschaftlichen Versorgungsforschung bei Menschen mit Migrationshintergrund: Möglichkeiten und Grenzen: Potential and Limitations. Gesundheitswesen. 2012:371–8. doi:10.1055/s-0031-1280759.
11. Brzoska, P., Voigtländer, S., Reutin, B., Yilmaz-Aslan, Y., Barz, I., Starikow, K., Reiss, K., Dröge, A., Hinz, J., Exner, A., Striedelmeyer, L., Krupa, E., Spallek, J., Berg-Beckhoff, G., Schott, T., Razum, O. Rehabilitative Versorgung und gesundheitsbedingte Frühberentung von Personen mit Migrationshintergrund in Deutschland. 2010. Rehabilitative Versorgung und gesundheitsbedingte Frühberentung von Personen mit Migrationshintergrund in Deutschland. Accessed 1 Mar 2020.
12. Brzoska P, Voigtlander S, Spallek J, Razum O. Arbeitsunfälle, Berufskrankheiten und Erwerbsminderung bei Menschen mit Migrationshintergrund. In: Schott T, Razum O, editors. Migration und medizinische Rehabilitation. 1st ed.: Beltz Juventa Verlag; 2013. p. 49–61.
13. Yilmaz-Aslan Y, Brzoska P, Schott T, Razum O. Reha aus Sicht von türkischen Migrant(inn)en. In: Schott T, Razum O, editors. Migration und medizinische Rehabilitation. 1st ed.: Beltz Juventa Verlag; 2013. p. 195–201.
14. Reiss K, Yilmaz-Aslan Y, Reutin B, Barz I, Starikow K, Schott T, et al. Reha aus der Sicht von Aussiedler(inne)n. In: Schott T, Razum O, editors. Migration und medizinische Rehabilitation. 1st ed.: Beltz Juventa Verlag; 2013. p. 162–194.
15. Brzoska P, Voigtlander S, Spallek J, Razum O. Reha-Erfolg bei Migrant(inn)en: Herkunftsländer im Vergleich. In: Schott T, Razum O, editors. Migration und medizinische Rehabilitation. 1st ed.: Beltz Juventa Verlag; 2013. p. 105–110.
16. Brzoska P, Voigtlander S, Spallek J, Razum O. Utilization and effectiveness of medical rehabilitation in foreign nationals residing in Germany. European Journal of Epidemiology. 2010;25:651–60. doi:10.1007/s10654-010-9468-y.

17. Voigtländer S, Brzoska P, Spallek J, Exner AK, Razum O. Die Inanspruchnahme medizinischer Rehabilitation bei Menschen mit Migrationshintergrund. In: Schott T, Razum O, editors. Migration und medizinische Rehabilitation. 1st ed.: Beltz Juventa Verlag; 2013.
18. Yilmaz-Aslan Y, Brzoska P, Schott T, Razum O. Reha aus Sicht von türkischen Migrant(inn)en. In: Schott T, Razum O, editors. Migration und medizinische Rehabilitation. 1st ed.: Beltz Juventa Verlag; 2013. p. 162–194.
19. Erbstößer S, Zollmann P. Versorgungsunterschiede zwischen deutschen und ausländischen Rehabilitanden. RVaktuell. 2015;4:88–99.
20. Göbber J, Pfeiffer W, Winkler M, Kobelt A, Petermann F. Stationäre psychosomatische Rehabilitationsbehandlung von Patienten mit türkischem Migrationshintergrund: Spezielle Herausforderungen und Ergebnisse der Behandlung. Zeitschrift für Psychiatrie, Psychologie und Psychotherapie. 2010;58:181–7. doi:10.1024/1661-4747.a000026.
21. Pfeiffer W, Göbber J, Winkler M, Kobelt A, Petermann F. Stationäre psychosomatische Rehabilitationsbehandlung von Versicherten mit Migrationshintergrund. Neue Ansätze in der psychosomatischen Rehabilitation. Regensburg: S. Roderer Verlag. 2010:49–70.
22. Gruner A, Oster J, Müller G, Wietersheim J von. Symptomatik, Krankheitsmodelle, Behandlungserleben und Effekte bei Patienten mit und ohne Migrationshintergrund in der psychosomatischen Rehabilitation. Zeitschrift fur Psychosomatische Medizin und Psychotherapie. 2012;58:385–93. doi:10.13109/zptm.2012.58.4.385.
23. Höhne A. Erwerbsminderungsrenten und medizinische Rehabilitation in Deutschland unter Berücksichtigung des Migrationshintergrunds: 12. bundesweiter Kongress Armut und Gesundheit. Berlin; 1./2. Dezember 2006.
24. Höhne A, Schubert M. Vom Healthy-migrant-Effekt zur gesundheitsbedingten Frühberentung. Erwerbsminderungsrenten bei Migranten in Deutschland. In: Deutsche Rentenversicherung Bund, editor. Etablierung und Weiterentwicklung. Bericht vom vierten Workshop des Forschungsdatenzentrums der Rentenversicherung (FDZ-RV) am 28. und 29. Juni 2007 im Wissenschaftszentrum Berlin für Sozialforschung (WZB). Berlin; 2007. p. 103–25 [DRV-Schriften; vol. 55] [cited 2020 Mar 6]. Available from: http://forschung.deutsche-rentenversicherung.de/FdzPortalWeb/getRessource.do?key=drv_band_55_s_103_126_hoene.schub.pdf. Accessed 6 March 2020
25. Höhne A, Behrens J, Schubert M, Schaepe C, Zimmermann M. Das Krankheitsspektrum von Erwerbsminderungsrentnern mit Migrationshintergrund. In: Deutsche Rentenversicherung Bund, editor. 16. Rehabilitationswissenschaftlichen Kolloquium: Gesund älter werden -mit Prävention und Rehabilitation vom 26. März bis 28. März 2007 in Berlin. Berlin; 2007. p. 207–9 [DRV-Schriften; vol. 72]. Available from: http://forschung.deutsche-rentenversicherung.de/ForschPortalWeb/ressource?key=tagungsband_16_reha-kolloqu.pdf. Accessed 6 March 2020
26. Jankowiak, S., Kaluscha, R., Krischak, G. Soziale Unterschiede bei der Beantragung und Inanspruchnahme von medizinischen und beruflichen Rehabilitationsleistungen. In: Deutsche Rentenversicherung Bund, editor. 27. Rehabilitationswissenschaftliches Kolloquium Deutscher Kongress für Rehabilitationsforschung: Rehabilitation bewegt! vom 26. bis 28. Februar 2018 in München. Berlin; 2018. p. 504–7 [DRV Schriften; vol. 113] [cited 2020 Mar 6]. Available from: http://forschung.deutsche-rentenversicherung.de/ForschPortalWeb/ressource?key=tagungsband_27_reha_kolloqu.pdf. Accessed 6 March 2020
27. Kaluscha R, Brzoska P, Jacobi E, Krischak G. Inanspruchnahme medizinischer Rehabilitation wegen psychischer Erkrankungen: Gibt es Unterschiede zwischen Menschen deutscher und ausländischer Staatsangehörigkeit? In: Rehabilitationswissenschaftliches Kolloquium: Nachhaltigkeit durch Vernetzung; 2011. p. 141–2 [DRV-Schriften; vol. 93] [cited 2020 Mar 6]. Available from: http://forschung.deutsche-rentenversicherung.de/ForschPortalWeb/ressource?key=tagungsband_20_reha_kolloqu.pdf. Accessed 6 March 2020
28. Kessemeier FM, Bassler M, Petermann F, Kobelt-Pönicke A. Therapeutische Allianz und Rehabilitationszufriedenheit von Menschen mit Migrationshintergrund in der psychosomatischen Rehabilitation – Analyse routinemäßig erhobener Daten. Physikalische Medizin, Rehabilitationsmedizin, Kurortmedizin. 2019;29:267–74. doi:10.1055/a-0862-0657.
29. Klinik für Rehabilitationsmedizin, Medizinische Hochschule Hannover (MHH), Ethnomedizinisches Zentrum e.V. (EMZ). MiMi-Reha: Implementierung und Evaluation eines Info-Angebotes für MigrantInnen zur medizinischen Reha auf Basis der ‚MiMi-Kampagnentechnologie': Das Gesundheitsprojekt Mit Migranten für Migranten - Abschlussbericht -; 2017.
30. Nowik D, Bergman J, Markin K, Reißmann L, Salman R, Gutenbrunner C. Veränderungen subjektiver Zugangsbarrieren und Antragsintention zur Rehabilitation von MigrantInnen – Abschließende Ergebnisse aus MiMi-Reha. In: Deutsche Rentenversicherung Bund, editor. 26. Rehabilitationswissenschaftliches Kolloquium Deutscher Kongress für Rehabilitationsforschung: Prävention und Rehabilitation in Zeiten der Globalisierung vom 20. bis 22. März 2017 in Frankfurt am Main; Sonderausgabe. Berlin; 2017. p. 86–8 [DRV-Schriften; vol. 111] [cited 2020 Mar 6]. Available from: http://forschung.deutsche-rentenversicherung.de/ForschPortalWeb/ressource?key=tagungsband_26_reha_kolloqu.pdf. Accessed 6 March 2020
31. Reissmann, L.-M., Schwarz, B., Markin, K., Salman, R., Gutenbrunner, C. Ein Wegweiser für Migranten in die medizinische Rehabilitation der Deutschen Rentenversicherung. In: Deutsche Rentenversicherung Bund, editor. 24. Rehabilitationswissenschaftliches Kolloquium Deutscher Kongress für Rehabilitationsforschung: Psychische Störungen – Herausforderungen für Prävention und Rehabilitation vom 16. bis 18. März 2015 in Augsburg; 2015. p. 191–2 [DRV Schriften; vol. 107] [cited 2020 Mar 6]. Available from: http://forschung.deutsche-rentenversicherung.de/ForschPortalWeb/ressource?key=tagungsband_24_reha_kolloqu.pdf. Accessed 6 March 2020

32. Schwarz B, Markin K, Salman R, Gutenbrunner C. Barriers for Migrants Regarding the Access to Medical Rehabilitation on Behalf of the German Pension Insurance. Rehabilitation (Stuttg). 2015;54:362–8. doi:10.1055/s-0041-108279.
33. Kohler M, Ziese T. Telefonischer Gesundheitssurvey des Robert-Koch-Instituts zu chronischen Krankheiten und ihren Bedingungen: Deskriptiver Ergebnisbericht. Berlin: Robert-Koch-Institut; 2004.
34. Maier C. Migration und rehabilitative Versorgung in Deutschland: Ein Vergleich der Inanspruchnahme von Leistungen der medizinischen Rehabilitation und eines Indikators für Rehabilitationserfolg zwischen Rehabilitanden türkischer und nicht-türkischer Abstammung; 2008.
35. Ritter S, Dannenmaier J, Jankowiak S, Kaluscha R, Krischak G. Implantation einer Hüft- oder Knietotalendoprothese und die Inanspruchnahme einer Anschlussrehabilitation. Rehabilitation. 2018;57:248–55. doi:10.1055/s-0043-102135.
36. Schröder CC, Dyck M, Breckenkamp J, Hasselhorn HM, Du Prel J-B. Utilisation of rehabilitation services for non-migrant and migrant groups of higher working age in Germany - results of the lidA cohort study. BMC Health Serv Res. 2020;20:31. doi:10.1186/s12913-019-4845-z.
37. Spallek L, Yılmaz-Aslan Y, Klein-Ellinghaus F, Gök Y, Zeeb H, Kolip P, et al. Deficits in psycho-oncological care among Turkish immigrant women with breast cancer in Germany – an interview study. IJPR. 2017;1:1–10. doi:10.14302/issn.2574-612X.ijpr-17-1746.
38. Yilmaz-Aslan Y, Spallek L, Gök Y, Kolip P, Spallek J. Krankheitsvorstellungen und Behandlungserwartungen nach der Diagnose Brustkrebs: Die besondere Situation türkischer Frauen. Dresden; 20.-22. November.
39. Zollmann P, Pimmer V, Rose AD, Erbstösser S. Comparison of Psychosomatic Rehabilitation for German and Foreign Patients. Rehabilitation (Stuttg). 2016;55:357–68. doi:10.1055/s-0042-120085.
